# An automated hybrid deep learning framework for paddy leaf disease identification and classification

**DOI:** 10.1038/s41598-025-08071-6

**Published:** 2025-07-24

**Authors:** Chatla Subbarayudu, Mohan Kubendiran

**Affiliations:** School of Computer Science and Engineering, Vellore Institute of Technology, Vellore, 632 014 India

**Keywords:** Paddy Plant Leaf, Deep Learning (DL), Adaptive Thresholding, Genghis Khan Shark (GKSO) Algorithm, Simulated Annealing (SA), CatBoost Classifier, k-means, Environmental sciences, Computational science

## Abstract

In India, agriculture remains the primary source of livelihood for many people. Pathogen attacks in crops and plants significantly diminish both the yield and quality of production, leading to financial losses. As a result, identifying diseases in crops is highly important. As the population grows, the demand for rice also rises. Therefore, disease management is vital in rice cultivation, and rapid identification of rice diseases is critical for timely pesticide application and effective control. Consequently, there is a need to boost agricultural productivity by adopting new technologies. Deep learning is a popular area of research in various fields. This research aims to design and propose a new automated model using a deep learning model for the disease identification and categorization of paddy leaves. The system follows a structured workflow comprising several stages: image acquisition, pre-processing, feature extraction, feature selection, and classification. Images of paddy leaves were obtained from the paddy doctor dataset hosted on Kaggle. The data is pre-processed by choosing the RoIs, labelling, enhancement, and segmentation using adaptive thresholding and grouped using K-means clustering. The MobileNetV3 model, a pre-trained transfer learning approach, extracted colour, shape, and texture features. The vital features are selected using the hybrid Genghis Khan Shark Optimization (GKSO) with Simulated Annealing (SA) algorithm. The chosen features are subsequently fed into the CatBoost for disease classification. The deep learning techniques introduced for disease identification and classification have been compared with various conventional classifiers, and the system’s performance has been validated using metrics such as accuracy, sensitivity, and F1-score. Performance investigations prove that the technique efficiently yields a higher accuracy of 98.52%, outperforming state-of-the-art techniques.

## Introduction

Nowadays, agriculture serves as a cornerstone for addressing the nutritional needs of an expanding population. Agriculture, fisheries, and forestry sectors contribute approximately 18% to the GDP. However, the share of agricultural output in the national GDP has been steadily declining over the years^1^. More than three billion people depend on rice as their primary source of sustenance. However, the term “rice” is quite general, as there are many different varieties cultivated across the globe^2^. As a Kharif crop, rice grows well in warm, humid climates and waterlogged conditions, which makes it vulnerable to various diseases. These diseases can be divided into two main categories according to their causes.

The first type is parasitic diseases, triggered by living organisms such as harmful organisms, pests, and weeds. Pathogens like fungi, bacteria, and viruses are responsible for a broad spectrum of infections in rice. Some of these diseases can significantly reduce crop yields. The second type is non-parasitic diseases, caused by environmental factors like extreme temperatures, radiation, nutrient deficiencies, or water-related issues. Such abiotic diseases include straight head, cold injury, white tip, panicle blight, alkalinity, and bronzing. The main problem is the need for regular plant monitoring. Additional factors could involve farmers’ inexperience, who may overlook important details or need more knowledge about diseases that impact plants and their seasonal patterns. These diseases can strike any time, but consistently tracking plant growth and development stages can help control their spread. Numerous pathogens, including viruses, bacteria, and fungi pose a substantial risk to rice production by causing illnesses like sheath rot, bacterial leaf blight, leaf folder, blast disease, sheath blight, brown leaf spot, and hispa^3,4^. Implementing corrective actions, such as applying fungicides in the early stages, can help prevent significant losses^5^. Without proper identification, applying pesticides becomes ineffective. This issue has inspired us to pursue research on identifying rice diseases prevalent in India. Rice plant infections may be categorized according to the origin of the infection or the specific plant part impacted. Symptoms may appear on the rice plant’s leaves, stems, panicles, or roots. These infections can lead to significant crop losses, with damage ranging from 30 to 100% of the yield^6^.

Detection of plant diseases at an early stage requires arduous effort^7^. Traditionally, farmers rely on manual methods, using their knowledge and visual observations to detect diseases in rice crops. However, this approach could be more efficient, time-consuming, and prone to errors^8^. It is even more crucial for experts to recognize the cumbersome disease and quantify it in the case of large paddy fields. Technological advancements, like ICT and IoT, have enabled the integration of real-time monitoring and disease detection, classification, and ramifications to a greater extent^9,10^. To assist this, ML and DL techniques have enabled a wide-open platform to detect, classify, and achieve accurate and timely diagnosis, especially by using DCNNs^11,12^. Though DCNN can handle large quantities of data, the model’s performance has been expanded even further using transfer learning architectures^13^. Feature extraction techniques help to identify various patterns, groups, and essential features that contain raw information and provide an understanding of disease detection and classification^14^. Existing methods based on C-Bi-LSTM suffer from less accuracy and suffers from unbalanced distribution of data^15^, RNN-LSTM^16^ suffers from low accuracy due to concealment of CNN methods, barouta feature selection algorithm^17^ suffers from high computation cost due to big data. Though different ML, DL, and optimization techniques are utilized, these techniques still suffer from vanishing gradients and accidental errors while training the model. Most optimization algorithms pose a limitation by converging to the local optima. Additionally, due to the high amount of data, the computational complexity leads to high computational time. This highlights the necessity of developing a novel automated rice foliage disease detection and categorization framework utilizing deep learning. Such a system would aid farmers by offering a cost-effective, lightweight computational model that delivers optimal performance.

### Motivation

The motivation behind detecting multiple diseases on the same leaf of paddy plants using deep learning stems from the critical need to enhance agricultural disease management. It is identified that only 20–30% of farmers in India have the knowledge and access to disease detection and classification methods. Traditional methods need help to accurately identify and differentiate between multiple coexisting diseases, leading to misdiagnosis, ineffective treatments, and significant crop losses. This provokes the need to design a new automated paddy leaf illness detection and classification method powered by deep learning, which would provide optimized performance and better adoption, thereby incrementing the productivity of the paddy crop, benefitting the farmers.

### Major contributions

The proposed automated hybrid system utilizes deep learning techniques for detecting and classifying paddy leaf diseases. It involves the use of the hybrid MobileNetV3^18^, a transfer learning CNN model, and GKSO-SA^19^, followed by classification using the CatBoost classifier^20^. The proposed disease detection and classification model involves the following significant contributions:

- Farmers lack knowledge and awareness of modern technologies to detect and recognize paddy leaf diseases. To assist them with a new automated hybrid MobileNetV3, a transfer learning CNN model along with GKSO-SA is utilized. This alleviates the need to wait for an expert, thereby allowing the farmers themselves to assess the corresponding paddy leaf diseases.
- The proposed work involves the extraction of RoIs, data labelling, resizing, filtering, augmentation, and segmentation using adaptive thresholding and k-means clustering techniques for image pre-processing.
- The features are then classified using the CatBoost classifier, which achieves the finest classification of all features, thereby liberating the selected features. The significant advantage of the CatBoost classifier is that it analyzes the image data with categorical labels, thereby providing a clear-cut analysis of the relationship between the features.
- An analysis of performance was conducted on the Google Colab framework using Python, demonstrating superior results over existing methods for detecting and classifying paddy leaf diseases, with an accuracy of 98.52%, surpassing traditional algorithms.

### Organization of the research work

The proposed work begins with Section. 1, which provides the introduction, motivation, and outlines the significant contributions towards the detection and categorization of paddy leaf diseases. This is followed by section. 2 "Related works" (related work), which reviews various studies conducted by researchers on paddy leaf disease detection and classification framework along with their solutions, benefits, and limitations. Section. 3 "Materials and methods" covers the materials and methods, offering a detailed discussion that leads into section. 4 "Proposed methodology", which presents the proposed methodology. Next, section. 5 "Experimentation" presents the experimental analysis. Section. 6 "Result analysis and discussion" discusses the results and discussion, and finally, section. 7 "Conclusion and future work" provides the conclusion and outlines direction for future work. Table 1 provides the chronicle of abbreviations utilized in the proposed work.

**Table 1:** Chronicles of abbreviations.

Acronym	Abbreviation
2D, 3D	Two-Dimensional, Three-Dimensional
ARFA	Adaptive Red Fox Algorithm
Bi-GRU	Bi-directional Gated Recurrent Unit
CBAM	Convolutional Block Attention Module
CNN	Convolutional Neural Network
CSP	Cross-Stage Partial
CV	Computer Vision
DCNN	Deep Convolutional Neural Network
DenseNet	Dense Neural Network
DenseNet	Dense Neural Network
DL	Deep Learning
DMCNN	Deep Multi-Scale Convolutional Neural Networks
FoR	False Omission Rate
FPR	False Positive Rate
GANs	Generative Adversarial Networks
GCL	Generative Adversarial Networks, Convolutional Neural Networks, Long-term short-term memory
GDP	Gross Domestic Product
GKSO	Genghis Khan Shark Optimization
SA	Simulated Annealing
GPU	Gross-Processing Unit
HGB	Histogram Gradient Boosting
ICT	Information and Communication Technology
IoT	Internet of Things
KNN	K-Nearest Neighbour
LDA	Linear Discriminant Analysis
MBi-LSTM	Modified Bi-Long Short-Term Memory
ML	Machine Learning
RegNet	Regulated Neural Network
RGB	Red Green Blue
RoI	Region of Interest
RoIs	Regions of Interest
SVM	Support Vector Machine
SV-RFE	Support Vector Machine-Recursive Feature Elimination
TLO	Teaching–Learning Optimization
VGG	Visual Geometry Group
XGB	Extreme Gradient Boosting
YOLO	You Only Look Once

## Related works

In agriculture, the application of image processing and pattern recognition tools to detect and diagnose plant diseases is growing. Computer vision systems, in particular, have played a key role in creating tools capable of accurately diagnosing and detecting disease symptoms in crops such as paddy. Recent developments in deep learning have empowered researchers to make significant strides in recognizing and detecting diseases and pests on plant leaves from images. These innovations assist farmers in identifying and managing diseases early, which helps to minimize crop losses and enhance overall productivity.

Bola et al.^21^ introduced a hybrid model built on traditional ML and DL techniques for identifying diseases of multiple crops, specifically in corn, rice, and wheat. This study compared the effectiveness of various methods to recognize illnesses in wheat, corn, and rice. As a result, the combination of DenseNet201 deep features with an SVM exhibited outstanding performance, reaching an exceptional accuracy of 87.23% with only 20.2 million parameters. The model was rigorously evaluated utilizing precision, recall, F1 score, and total accuracy metrics. It efficiently distinguishes between healthy and infected plants for each crop, attaining accuracy rates of 99.82% for corn, 98.75% for wheat, and 84.15% for rice.

Furthermore, integrating real-time data from crop fields with images can significantly improve the accuracy of disease classification and identification. Omer et al.^22^ suggested an algorithm for detecting diseases and pests on cucumber leaves using an enhanced YOLO v5 model, where the CSP module in the backbone was replaced with the CBAM. They developed a dataset consisting of 3,057 real-world images of cucumber leaf diseases. The modified method attained a superior appreciation accuracy of 80.10%, outperforming the original YOLO v5. Future work will refine the network model structure to improve cucumber leaf disease and pest detection performance. Dubey et al.^23^ designed a deep-learning model for the automated detection and classification of leaf conditions. Feature selection was initially carried out by combining SV-RFE and ARFA. The selected features were then used in an MBi-LSTM classifier to categorize the visuals as either Tungro, Bacterial Leaf Blight, Blast disease, or healthy. The proposed feature selection approach outperformed individual models, achieving a high accuracy of 97.16% based on various classification metrics compared to state-of-the-art techniques. However, this method is limited to diagnosing specific crop diseases. Elfatimi et al.^24^ projected a CNN method for detecting and preventing ten common tomato leaf diseases using DMCNN. They constructed a dataset of 11,000 images of tomato leaf diseases sourced from Kaggle. The recommended model attained a high accuracy of 99.1%, demonstrating its effectiveness in the early detection and prevention of plant leaf diseases. Future work aims to apply the model to other crops and explore transfer learning, although its practical implementation in precision agriculture has yet to be tested.

Wang et al.^25^ introduced a CNN model to classify seven common apple leaf diseases using an optimized, lightweight RegNet model. This model utilized a dataset of 2,609 images of apple leaf diseases sourced from Kaggle. The model demonstrated good generalization for Dataset1 and Dataset2, achieving testing accuracies of 93.85% and 99.23%, respectively. The model tested on Dataset 1 attained an accuracy of 93.85%, with 24 samples misclassified. Despite efforts, the model needed help to improve the testing accuracy. RegNet-trained models demonstrated superior robustness and accuracy, identifying various diseases in leaf images despite complex visual interference. Stephen et al.^26^ analyzed a new method utilizing 3D and 2D Convolutional Neural Networks for feature extraction in predicting rice leaf disease. They collected 5,200 images of rice leaf diseases to address the problem faced by farmers, who typically rely on visual examination, which is susceptible to errors, labor-intensive, and can result in significant yield loss due to misjudgment in detecting three varieties of paddy leaf diseases. The concept of rice leaf prediction was implemented using Google Colab with the IBS-optimized DGAN. The proposed IBSGAN architecture model accomplished a high accuracy of 98.7%, outperforming other established methods like XGBoost, transfer learning approaches, and support vector machines. The advantage of the IBS algorithm is that it primarily addresses instability and overfitting problems commonly encountered in DGAN.

Future studies will examine a broader range of leaf characteristics and address additional diseases in rice plants. Singh et al.^27^ discussed the automatic identification of fungal blast leaf sickness in paddy crops. The proposed approach utilized five deep transfer learning algorithms based on convolutional neural networks for binary classification, namely InceptionV1, AlexNet, Xception, LeNet, and VGG-16 models. The algorithm performance evaluation showed that AlexNet achieved the highest accuracy for binary classification, with an average of 98.7%. The evaluation parameters included accuracy, sensitivity, specificity, F1 score, FDR, and FOR. A dataset containing 6,300 images of rice leaves affected by fungal blast disease was gathered in real time. The model’s scope could be expanded to focus on more diseases in the same crop and could also be applied to detect diseases in other crops and fruit plants. Integrating it with IoT, computer vision, and robotics could improve its effectiveness. Dubey and Choubey^28^ explored a method for classifying rice leaf diseases, employing a highly efficient adaptive feature selection strategy.

This approach involves the creation of a model by the authors to identify four distinct diseases affecting the leaves of paddy plants: blast disease, bacterial leaf blight, Tungro, or healthy for image acquisition. The model consists of four stages: pre-processing, feature extraction, selection, and classification. With this approach, the input image was directed to the pre-processing stage, where it was converted to RGB color, and the noise was reduced using a median filter. The results showed that, compared to other state-of-the-art models, the proposed deep learning technique achieved an accuracy rate of 98.86%. Dogra et al.^29^ suggested a model for detecting rice leaf diseases using automated CNNs. Their approach specifically focused on identifying brown spot disease in the context of intelligent agriculture. The study contrasted the traditional, time-consuming manual process of disease identification with an automated system utilizing deep learning CNN models. The automated approach reached the highest accuracy. Four key metrics, sensitivity, specificity, precision, and F1-score, were evaluated, with VGG-19 achieving the best accuracy of 93.0%. Transfer learning methodologies were employed to improve prediction accuracy for more diverse datasets and additional rice leaf diseases, which enhanced accuracy and simplified the model training process. Bharanidharan et al.^30^ developed a machine-learning approach to categorize five types of paddy diseases: bacterial leaf blight, hispa, leaf folder, brown leaf spot, and rice blast. The model detects paddy leaf diseases using a filter-based feature transformation method, utilizing a dataset of 636 images. Each thermal image was used to extract seven statistical features and an additional seven Box-Cox transformed statistical attributes. Four machine learning techniques were tested: KNN classifier, Random Forest classifier, LDA classifier, and HGB classifier. By applying the suggested feature transformation method, the K-Nearest Neighbor classifier achieved the highest accuracy, reaching 90%.

Latif et al.^31^ developed a deep learning-based approach, modifying a VGG19 transfer learning method to accurately classify and diagnose six categories, such as healthy rice plant leaves and five diseases: bacterial leaf blight, brown spots, leaf blasts, leaf scalds, and narrow brown spots. The system was trained on a non-normalized augmented dataset, achieving a high accuracy of 96.08%. Future work will concentrate on developing an IoT system integrated with deep learning and powered by drones, to be tested under real-world, real-time conditions. Additionally, the aim is to improve deep learning approaches for identifying all rice leaf diseases and expand the study to cover other significant plant diseases in the agricultural sector. Sethy et al.^32^ presented a system that used 5,932 field images representing four categories of rice leaf diseases: blast, bacterial blight, tungro, and brown spot. The system incorporated an evaluation of the performance of 11 CNN models, utilizing a transfer learning technique along with deep feature extraction and SVM for classification. In this method, ResNet50 and SVM demonstrated the highest effectiveness as a classification model, attaining an F1-score of 0.9838 for deep feature-based identification of diseases in rice leaves. The scope of this research could be expanded by incorporating a broader range of rice diseases and further optimizing the CNN model for enhanced performance.

Additionally, future work will focus on developing an integrated application suitable for low-end devices. A limitation of this study is the potential need for further refinement to ensure the model’s robustness across various environments and device constraints. Debnath and Saha^33^ examined detecting rice leaf diseases using real-time image data, focusing on IoT-based smart farming with a CNN model for detecting and categorizing diseases. A primary advancement of their developed model lies in its capacity for early detection of Brown Spot disease in rice paddies, using a tailored image-processing tool for pre-processing and feature extraction. The model was trained on a dataset manually collected from rice fields and achieved a high accuracy of 97.701%. This approach could be extended to identify multiple disease types and assess the severity of crop infestations. Lamba et al.^34^ proposed the categorization of rice plant leaf diseases, including bacterial blight, leaf smut, and rice blast, based on machine learning approaches, which include GCLSTM networks, CNN, LSTM, and GAN. While the current research has achieved 97% accuracy in identifying these diseases, there still needs to be a gap in detecting disease severity levels. This study is restricted to disease identification without considering the varying degrees of severity for the three paddy diseases under investigation. Future research could focus on developing methods to classify the severity of these diseases.

Upadhyay and Gupta^35^ introduced a modified ResNeXt-based deep learning model for detecting fungal diseases across multiple fruit crops apple, custard apple, and guava collected from diverse regions. The approach outperformed prior methods such as Inception-v3 and ResNet, achieving 98.92% accuracy using a heterogeneous dataset of 14,408 images. Although the model excels in disease identification, it does not address the classification of disease severity levels. Future research should aim to incorporate severity estimation to support better decision-making in crop management. Upadhyay and Gupta^36^ presented a meta-learning-based approach for estimating mango crop maturity using few-shot learning and DenseNet-121 architecture. The framework integrates Faster R-CNN for mango segmentation, followed by feature extraction and classification using cosine similarity. Their method achieved 83.65% accuracy in a 5-shot classification setting, outperforming conventional models like MAML and D-CNN. Despite its effectiveness, the study focused solely on binary classification without assessing gradual maturity stages. Future research could explore multi-level maturity estimation to support more nuanced harvesting strategies. Upadhyay and Gupta^37^ proposed a CNN-based framework incorporating data augmentation and VGG19 for detecting potato leaf diseases such as early and late blight. The model achieved a classification accuracy of 99.2% on a dataset of 2,152 images. Multiple classifiers were evaluated, with logistic regression outperforming others when combined with VGG19-extracted features. While the approach is highly effective in identifying diseases, it focuses only on classification and does not address the varying severity levels of infection. Future studies could explore severity estimation to support more precise treatment planning. Upadhyay and Gupta^38^ developed an enhanced ResNeXt-based deep learning model for diagnosing fungal diseases in apple crops. Using a benchmark dataset of 9,395 images spanning four disease categories (scab, rot, rust, and healthy), the model incorporated preprocessing, segmentation with GrabCut, and a modified ResNeXt architecture with reduced kernel sizes. The approach achieved 98.94% accuracy, outperforming standard ResNet and Inception-v3. While the study demonstrated high precision in disease identification, it was limited to classification and did not address severity assessment. Future studies could expand to quantify disease severity and adapt the model to other crop types. Upadhyay and Gupta^39^ conducted a comprehensive survey on the use of artificial intelligence in agricultural crop disease detection, reviewing over a decade of literature. The study examined a wide range of AI models, including SVM, ANN, CNN, and hybrid approaches, across multiple crops such as rice, tomato, maize, and apple. While the survey highlighted significant advancements in classification accuracy some exceeding 99% it also noted a research gap in severity estimation and real-time implementation. Future work could focus on integrating AI techniques for early-stage severity assessment and deploying scalable, field-ready solutions. Table 2 presents a comparison of different studies on paddy leaf diseases.

**Table 2:** Comparative evaluation of different research works pertaining to paddy leaf disease.

Refs	Problem Addressed	Solution	Benefits	Limitations
^21^	Identification of leaf diseases across multiple crops, including corn, rice, and wheat	DenseNet201 + SVM	99.82%, 98.75%, 84.15%	Accuracy needs to be improved
^22^	Detection of cucumber leaf diseases and pests using deep learning	YOLOv51 model	80.10%	The model architecture requires enhancement to improve the system’s performance
^23^	Paddy plant leaf disease classification	DL/MBi-LSTM/ SV-RFE and ARFA	97.16%	This technique is limited to diagnosing certain crop diseases and may not apply universally
^24^	Automated categorization of multi-class leaf diseases in tomatoes	Deep multi-level convolutional neural network (DMCNN)	99.10%	Practical deployment in precision agriculture remains untested
^25^	Identification of Multiple Diseases in Apple Leaf	RegNet DCNN	93.85% & 99.23%	The accuracy achieved is 93.85%, with 24 samples misclassified
^26^	Rice leaf disease prediction	IBS-optimized DGAN	98.70%	Multiclass and a more comprehensive range of leaf features must be considered
^27^	Automated detection of blast disease in paddy crop	AlexNet, LeNet, VGG 16	98.7%, 98.2%, 97.8%	Real-time testing has to be conducted
^28^	Paddy plant leaf disease classification	SV-RFE and ARO and ABi-LSTM	98.86%	It suffers from generalizability
^29^	Detection of brown spot rice leaf disease	CNN and Visual Geometry Group (VGG)19	93.00%	The proposed method focuses on a single disease in paddy crops. However, it should also be expanded to cover other crops and improve accuracy
^30^	Multiclass paddy disease detection	ML (KNN, Random Forest, LDA, Histogram Gradient Boosting)	90%	Transformer models have yet to be investigated for other crops
^31^	Detection of Rice Plant Diseases	Deep Convolutional Neural Network (DCNN)	96.08%	Lack of validation is still a question
^32^	Rice leaf disease identification	ResNet50 plus SVM	F1-score 98.38%	Focused on a narrow range of rice diseases
^33^	Early disease detection in rice paddy	IoT-based intelligent farming using CNN	97.70%	Severity estimation remains a challenge
^34^	Paddy plant leaf disease classification	GCL	97%	Severity estimation remains a challenge
^35^	Fungal disease detection across multiple crops	Modified ResNeXt CNN	98.92%	Severity estimation remains a challenge
^36^	Mango crop maturity estimation	Meta-Learning with DenseNet-121 Architecture	83.65%	Multi-level estimation remains a challenge in harvesting and requires improvements in accuracy
^37^	Potato leaf disease detection	Data Augmentation using VGG-19	99.2%	Severity estimation remains a challenge
^38^	Diagnosis of Fungal Diseases in Apple Crops	Enhanced ResNeXt Architecture	98.94%	Severity estimation remains a challenge, and there is a need to adapt the model to other crop types
^39^	Crop Disease Detection in Agriculture	AI Models Including SVM, ANN, and CNN	99%	Early-stage severity assessment continues to be a challenge

Table 2 shows that the existing methods suffer from high computational costs, and complex models are heavy, leading to decreased efficiency. To alleviate this problem, the proposed model focused on lightweight computations that would effectively benefit the farmers in performing disease detection and classification.

## Materials and methods

**Fig. 1:**
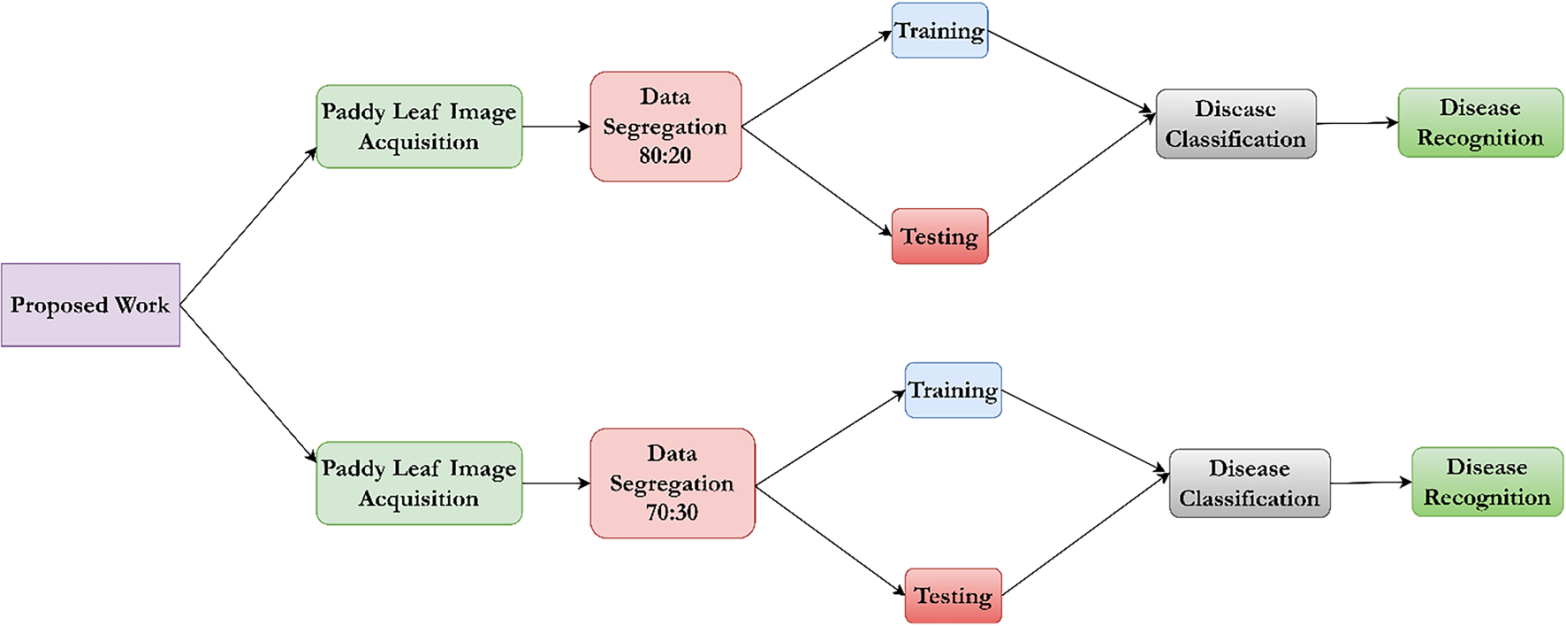
Proposed methodology.

The proposed automated paddy leaf disease detection and classification model involves various stages: image acquisition, image labelling, image augmentation, image segmentation, image feature extraction, image feature selection, and image feature classification. The doctor paddy dataset^40^ is utilized to carry out the proposed work. The dataset contains diseases, namely bacterial leaf blight^41^, bacterial leaf streak^42^, bacterial panicle blight^43^, blast^44^, brown spot^45^, dead heart^46^, downy mildew^47^, hispa^48^, and tungro^49^ where the research work has been focused on. Figure 1 provides the working methodology of the proposed new automated deep learning model for paddy leaf disease detection and classification.

The proposed work has been carried out by segregating the image data into 80:10:10 and 70:15:15. In the first phase, 80% of images are used for train the model, and the desired features are selected for classification by the classifier. The remaining untrained pictures of 10% are fed into the model during the testing and the remaining 10% for validation. In the second phase, 70% of images are used to train the model, and the desired features are obtained. They are fed into the classifier for classification. For testing, 15% of unknown images are tested, and the other 15% are utilized for validation. Each stage involves various processing methods, which are explained in detail as follows:

*Image data acquisition* Data collection is crucial in hoarding information from distinct sources^50^. Data being acquired enables businesses to gain meaningful insights utilizing ML models. The system will be trained and tested on thousands of images using the CV-based models. The AI model will utilize the dataset for classification and recognition^51^. *Image Data Augmentation* Image data enhancement is performed to prevent overfitting. Also, the issue of class imbalance or smaller datasets can be eradicated with the help of data augmentation. The proposed work involves data enhancement operations on images, such as flipping, cropping, rotation, geometric alterations, and colour enhancement^52^. *Image Data Labeling* Image data labelling involves tagging images for multi-class classification. Image data labelling is done for multi-class classification and bounding boxes^53^. *Image Pre-processing* The image collection should be pre-processed to eliminate noise, blurring, and low contrast has to be carried out^54^. *Image Segmentation* To improve the underlying deep learning model’s performance and computation complexity, the images are segmented. The proposed work involves various image segmentation approaches based on regions, edges, and clusters^55^. *Image Feature Extraction* Features are the pixels in the form of integer, float, or binary representation of numbers in an image. These pixels carry specialized information that is used for classification and recognition. The proposed work involves feature extraction based on color, text, and shape^56,57^. *Image Feature Classification* The categorization of image features is referred to as supervised classification, as the image data is labeled in the proposed study. The underlying deep learning model gets trained and tested with images, yielding the final classified output^58^.

### Image acquisition

**Fig. 2:**
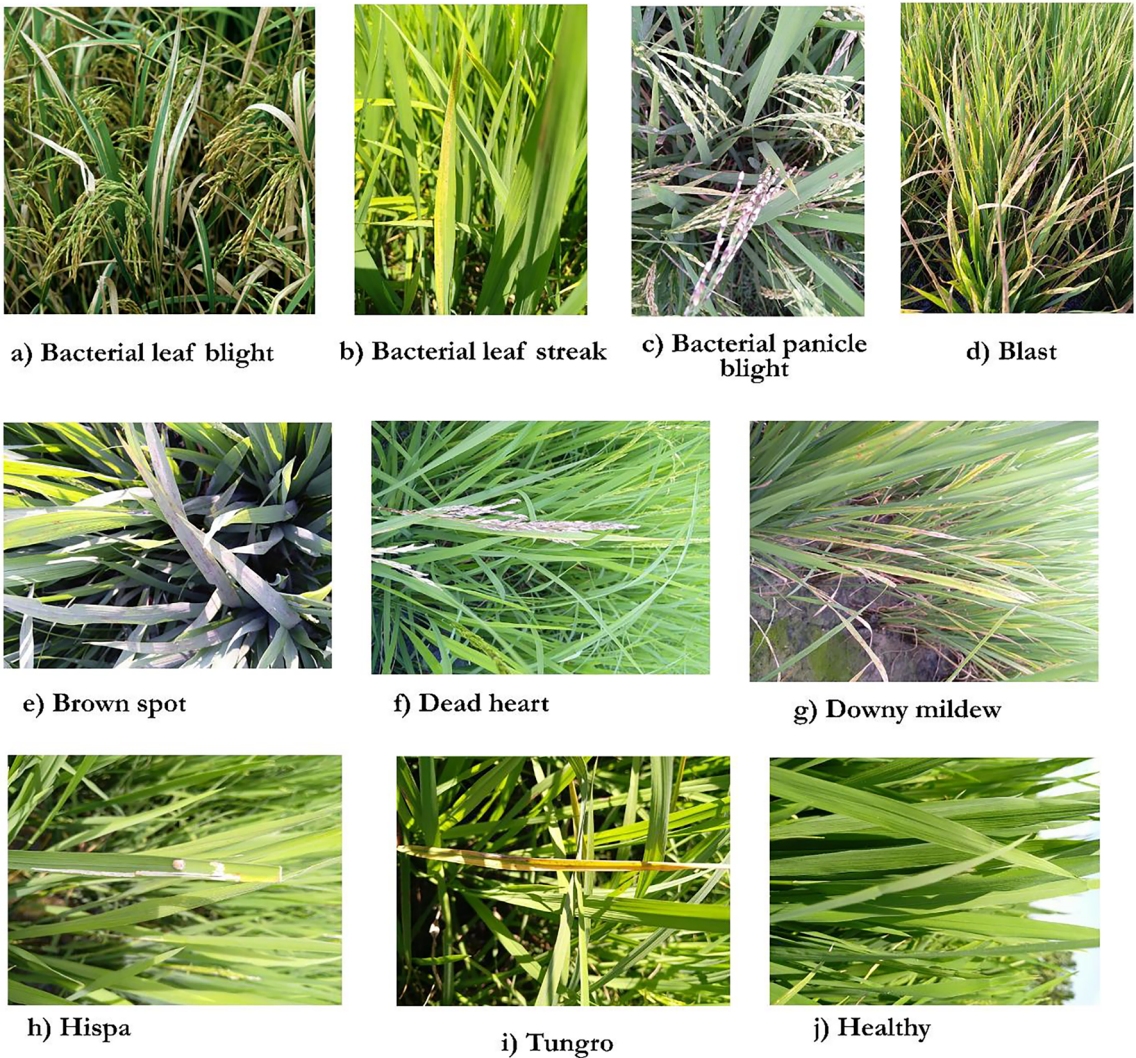
Diseases in paddy crops from the paddy doctor dataset.

The proposed work utilizes the Paddy Doctor dataset of 13,866 diseased and normal paddy images obtained from the Kaggle repository. A high-end GPU machine has been employed to train and test the deep learning model to process vast images. DCNN has been utilized to carry out image classification and recognition since it reduces the problem of dimensionality and offers high accuracy and faster computations. The training and testing have been conducted by splitting and distributing the images into epochs to optimize the effectiveness of the deep learning model. The virtual machine was created using Microsoft Azure based on Windows 11 pro 24H2 and supported by NVIDIA Tesla’s V100 GPU and virtual CPUs with 128 GB RAM^59,60^. (i) *Selection of RoIs* The disease-affected regions are cropped at the infected areas of an image in freehand to extract the RoIs. Cropping of the infected areas of an image facilitates better focus, achieving high efficiency, thereby enabling the model to concentrate on irrelevant pixels^61^. (ii)** Data labeling** The proposed work encompasses Azure Machine Learning Studio, which provides various data labelling tools with ML-assisted data labelling capability. Data labels are created using ML models that run in the background to speed up the process. In the case of the DL, the transfer learning technique jump-starts the training process by leveraging a pre-trained model where the data gets labelled^62,63^. Figure 2. provides the diseases in the paddy doctor dataset chosen for the proposed methodology.

### Image pre-processing

**Fig. 3:**
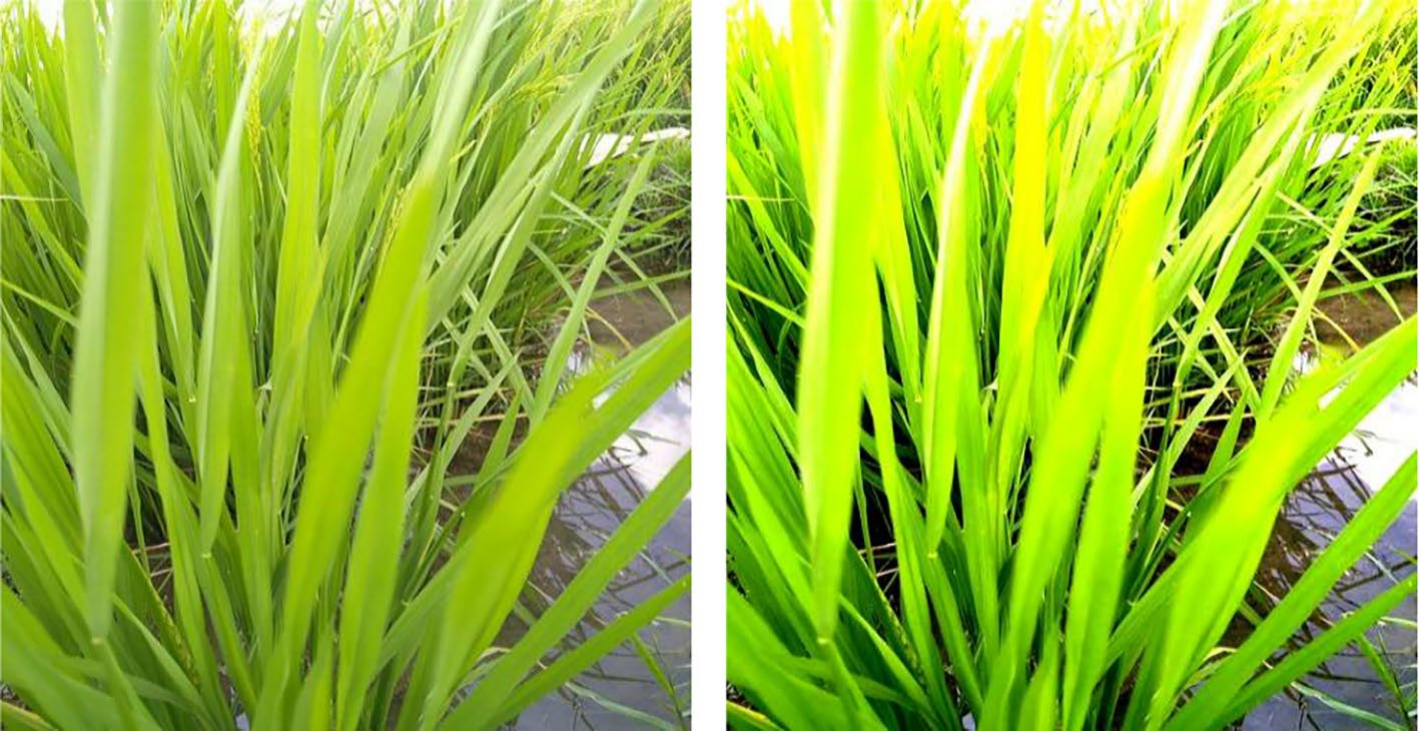
Image contrast enhancement operation.

**Fig. 4:**
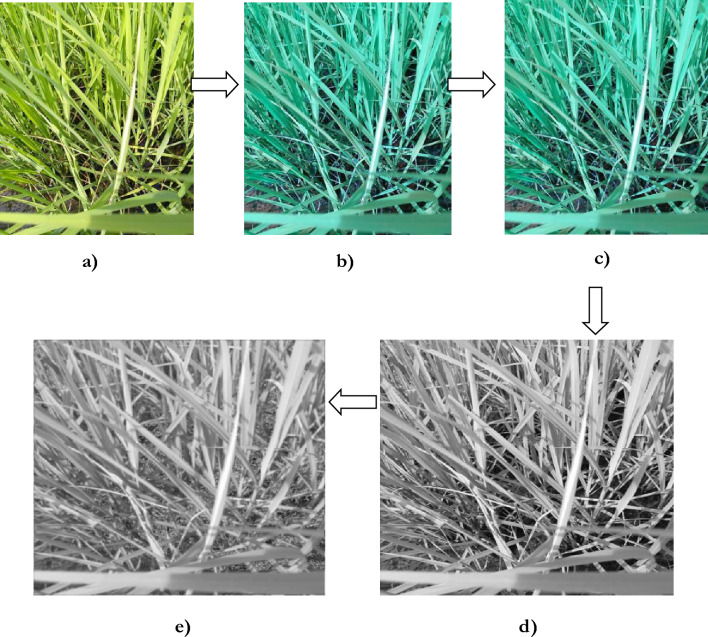
Image pre-processing operation.

Image pre-processing involves the following stages: image resizing, filtering, contrast enhancement, augmentation, segmentation, thresholding, edge detection, and color-based K-means clustering. (i) *Image resizing* The paddy images are adjusted to a uniform size, with a resolution of 1500 × 1500 pixels. Resizing the images helps minimize the memory storage requirements^64^. (ii) *Image filtering* Speckle noise is an inherent part of an image that is supposed to be removed for feature extraction. A wiener-frost filter is utilized to preserve the edge information and the feature retention^65–67^. The Wiener filter eliminates the noise with specks, where the speckling noise gets removed. Exponential weighting is utilized in a frost filter, which removes the additive noise. Thereby, the texture data of the image gets retained. (iii) *Color Contrast Enhancement* The primary purpose of performing contrast enhancement is to identify an image’s normal and diseased areas. Contrast enhancement takes the input as a grey scale and provides the output as a histogram-enhanced image^68^. Figure 3. provides the transformation of the image after performing image contrast enhancement operations. Figure 4. provides the transformation of the images during image pre-processing.

### Image augmentation

**Fig. 5:**
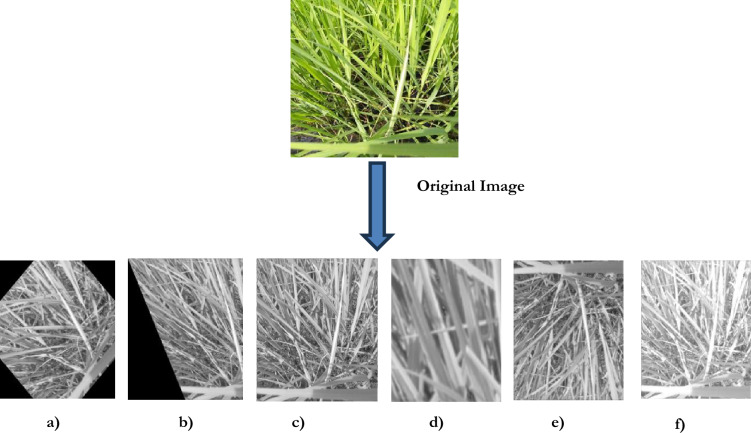
Augmentation operations.

Overfitting and underfitting significantly impact the performance of the underlying deep-learning technique^51^. Therefore, image augmentation has been performed to alleviate this. Although image augmentation expands the dataset size, it supports deep learning during the network training. The technique balances data by creating alternate versions of the same image^52^. Some of the image augmentation techniques employed in the proposed methodology include zooming, rotation, cropping, flipping, sheering, and enhancing the brightness of the pixels in an image^69^. Figure 5. provides the output of the various augmentation operations performed on a single image.

#### Image segmentation

Image segmentation is the process of grouping different pixels that contain important information^70^. The primary objective of image segmentation is to understand the visual information carried by the pixels and to achieve uniformity^71^. In the proposed methodology, three distinct methods are performed for segmenting the images: adaptive thresholding^72^, Prewitt edge detection^73^, and color fuzzy k-means clustering^74^.

#### Adaptive thresholding

**Fig. 6:**
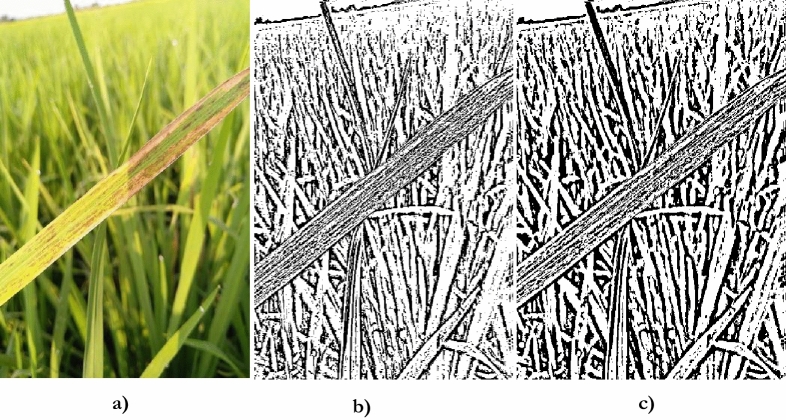
Adaptive thresholding operation.

Thresholding has been carried out to differentiate distinct objects in an image. Adaptive thresholding identifies the smaller regions of an image and ascertains the threshold for each area. Figure 6. provides the output obtained after adaptive thresholding.

#### Prewitt edge detection method

**Fig. 7:**
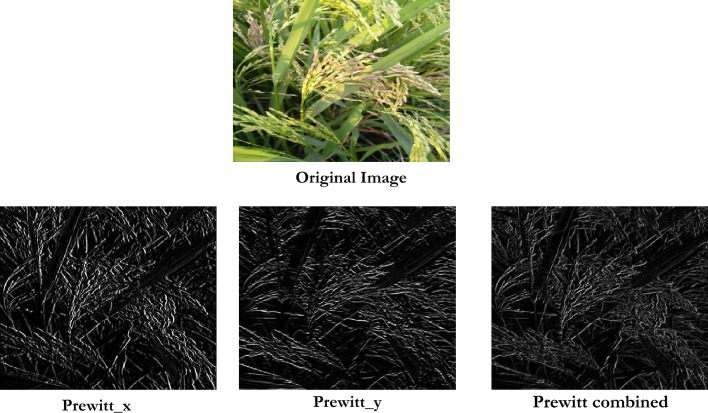
Prewitt edge detection operation.

Prewitt edge detection is utilized to identify the edge that preserves essential information. Convolution kernels are employed to calculate the gradient magnitude of the pixels in the image^75^. Prewitt utilizes horizontal and vertical kernels along directions to compute gradient magnitude^76^. Figure 7. provides the output obtained after prewitt edge detection.

#### Color fuzzy k-means clustering

Clustering involves grouping objects (pixels) in an image. It segregates the pixels that share similar or distinct properties and forms clusters. *K-means Clustering* works based on a group of randomly chosen centroids and finds an optimal computation for the placement of the centroids. The major disadvantage is that the effectiveness of clustering is directly proportional to the k-value, which is challenging^77^.

**Fig. 8:**
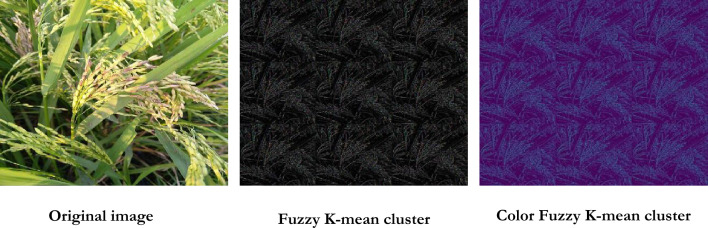
Color Fuzzy K-means clustering.

**Fig. 9:**
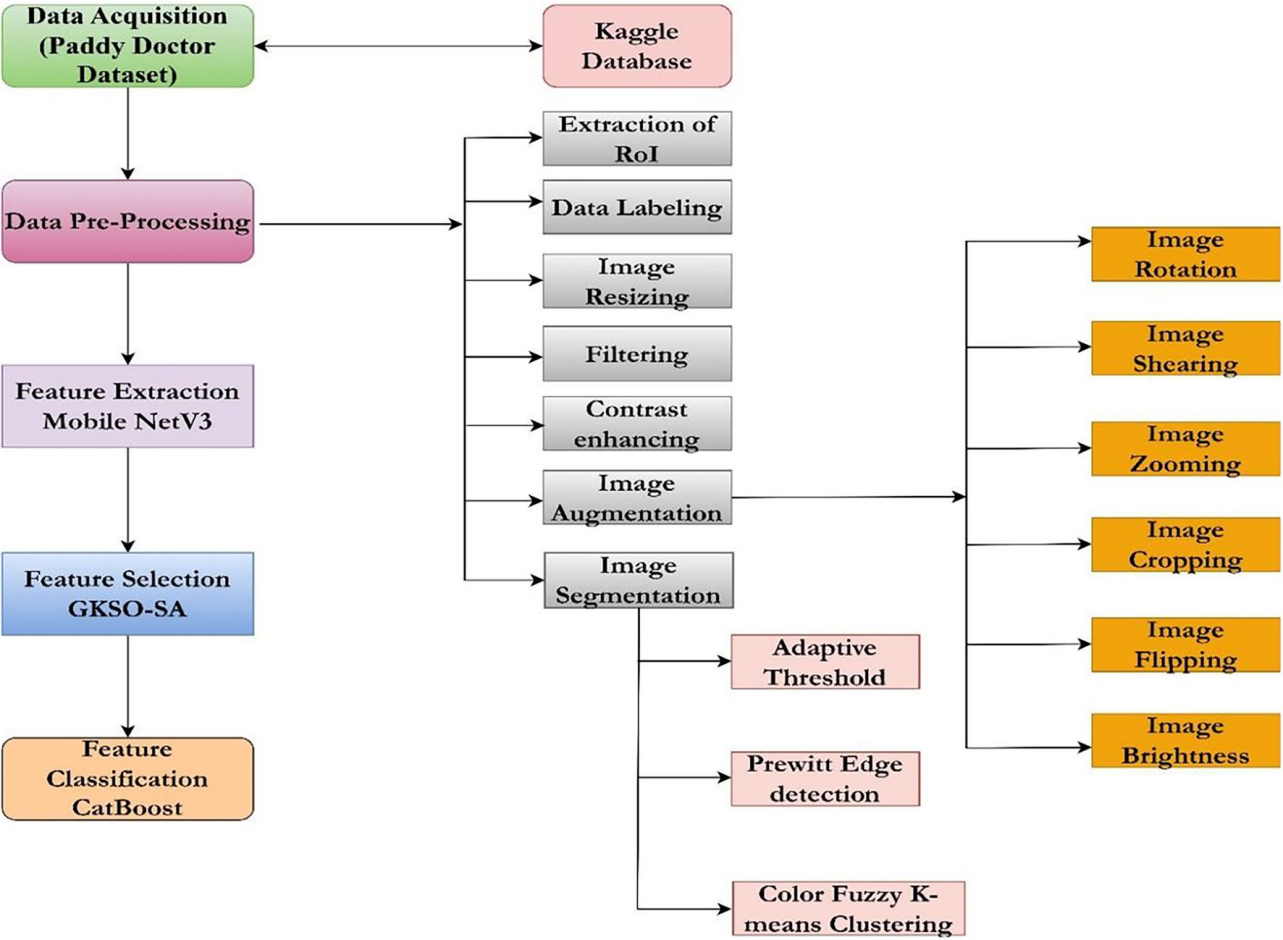
Proposed hybrid deep learning technique.

Figure 9 provides the output obtained after color fuzzy k-means clustering. Figure 8 shows the image clusters of distinct objects in violet color. The proposed color fuzzy k-means clustering technique yields good performance by segregating the pixels that carry information about both the foreground and background. The morphological activity over the image yields better segmentation precision with high computation speed^78^.

## Proposed methodology

The proposed methodology adopts a structured approach to detect and classify paddy leaf diseases using a hybrid deep learning model. It combines efficient feature selection with advanced classification methods to enhance overall accuracy.

### Proposed hybrid deep learning model with optimization algorithm

The proposed automated framework for paddy leaf disease detection and classification begins with preprocessing a Kaggle dataset comprising 13,866 images, covering nine disease categories and one healthy class. To enhance image quality and feature distinctiveness, images were initially resized to 1500 × 1500 pixels, denoised using the Wiener-Frost filter, and further processed through contrast enhancement and segmentation techniques such as adaptive thresholding, Prewitt edge detection, and fuzzy K-means clustering.

Feature extraction was performed using MobileNetV3^79^, a lightweight deep convolutional neural network based on transfer learning. Due to its efficient architecture and suitability for edge devices, MobileNetV3 effectively extracts features with reduced computational cost. Input images were resized to 224 × 224 pixels, and the model generated 128-dimensional feature vectors using depthwise separable convolution layers with ReLU and h-swish activation functions.

To refine the extracted features, a hybrid feature selection technique combining Genghis Khan Shark Optimization (GKSO)^80^ and Simulated Annealing (SA)^81^ was applied. GKSO mimics intelligent foraging behaviors to explore the feature space locally, while SA enhances global exploration by probabilistically accepting suboptimal solutions, thereby avoiding local optima. This complementary strategy enables adaptive and efficient selection of informative features.

The optimized feature subset was subsequently classified using the CatBoost algorithm^82^, an efficient gradient boosting framework on decision trees. It was configured with 1000 iterations, a tree depth of 6, a learning rate of 0.1, Logloss as the objective function, and a verbosity interval of 100. The model was trained for both binary (healthy/diseased) and multi-class (individual diseases) classification tasks.

All model components were fine-tuned using a grid search strategy. Specifically, MobileNetV3 was optimized with a batch size of 32, a learning rate of 0.0001, and the Adam optimizer, while GKSO-SA was set with a population size of 30, 1000 iterations, and a cooling rate of 0.95. The integrated MobileNetV3 + GKSO-SA + CatBoost model achieved a classification accuracy of 98.52%, demonstrating superior performance compared to conventional machine learning and deep learning models^83^. The proposed hybrid deep learning model with an optimization algorithm, as described in the proposed methodology, is depicted in Fig. 9.

#### Feature extraction using MobileNetV3

**Fig. 10:**
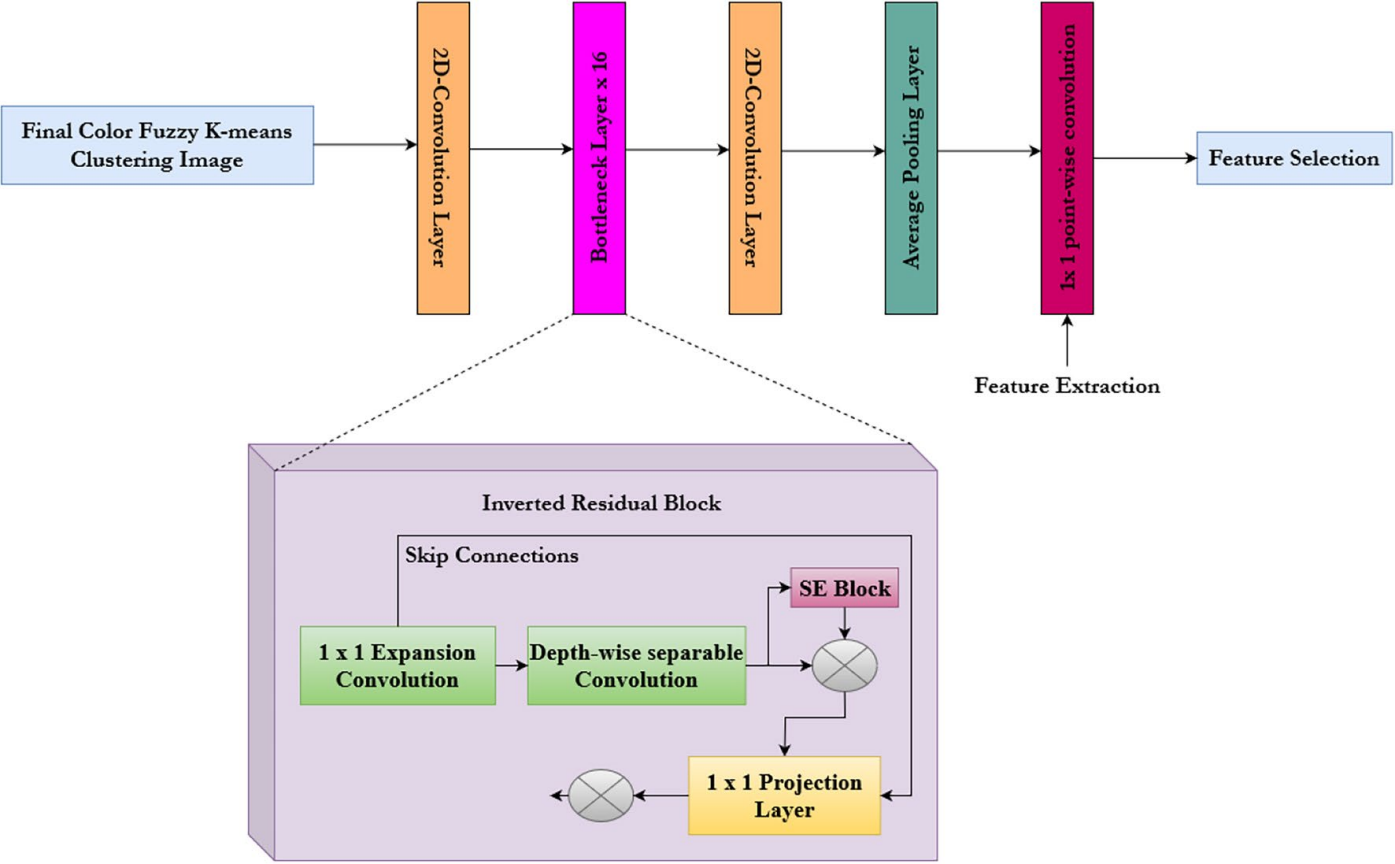
Overview of MobileNetV3 architecture.

To extract features for the proposed paddy disease classification, MobileNetV3, a deep learning algorithm based on transfer learning, has been used^84^. Leveraging the pre-trained weights on the Paddy Doctor dataset, the proposed model takes the image size of 224 × 224 and outputs a feature vector of size 128, which acts as an input to the feature selection process. The top layers of the mobilenetV3 have only been trained superseding by 1 × 1 point-wise convolution layers for feature extraction. The weights were adjusted during the fine-tuning of the network, consisting of two 2D convolution layers, 16 bottleneck layers, and an average pooling layer, depicted in Fig. 10.

**Fig. 11:**
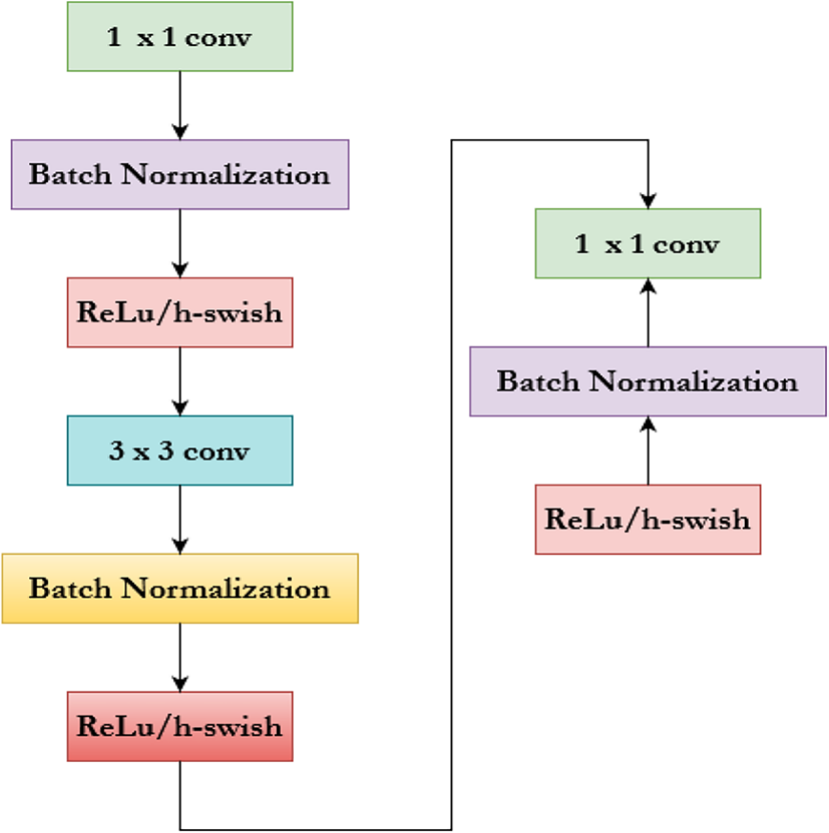
Components of depth-wise separable convolution layer.

The learned features are saved as vectors, representing the clustered input images. The inverted residual block includes a depth-wise convolution layer, serving as the core component of the training model. The depth-wise separable convolution layer includes a squeeze-and-excitation block to extract essential features on a per-channel basis. The depth-wise convolution layer utilized for feature extraction is 3 × 3. It supersedes the 1 × 1 point-wise convolution, which reduces the computational overhead while training the network in case of surplus images. To transform the output into a linear combination, 1 × 1 point-wise convolution was applied to each channel in the inverted residual block. This resembles that of an MLP. Figure 11. provides various components utilized inside the depth-wise separable convolution layer.

Two distinct activation functions, ReLu and h-swish^85,86^, are utilized, which significantly reduces the computational overhead incurred. The formula for the h-swish activation function is provided in Eq. (1) as follows.

1 $$h-swish=y*\sigma \left(y\right); \sigma \left(y\right)=\frac{ReLu 6\left(y+3\right)}{6}$$

From the Eq. (1) $\sigma \left(y\right)$ denotes the piece-wise linear hard analog function. The major advantage of using MobileNetV3 is the reduction in the representation space.

### Feature selection using GKSO-SA

**Fig. 12:**
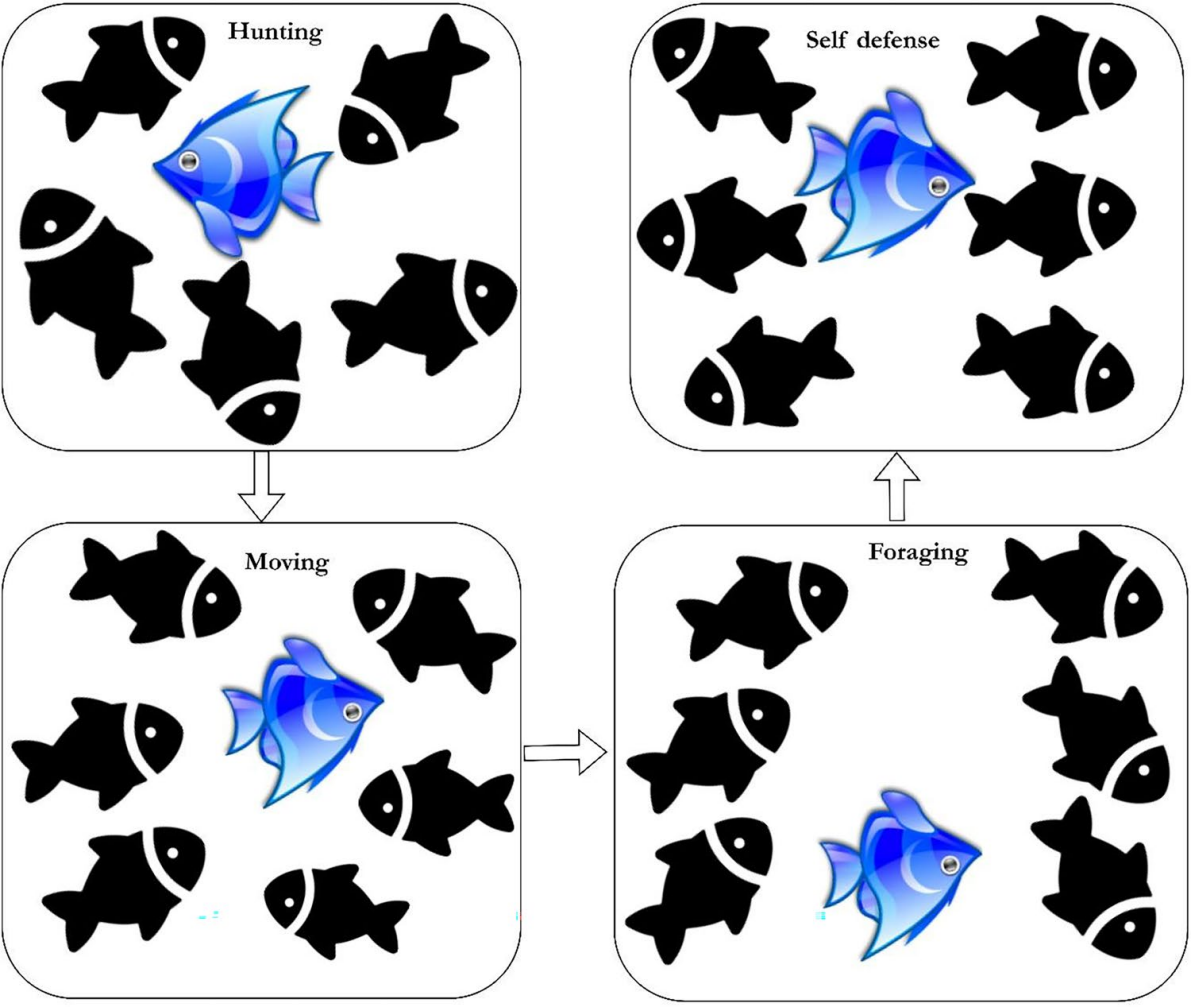
GKSO exploration & exploitation behaviour.

GKSO is a population-based optimization algorithm composed of four stages: hunting, movement, foraging, and self-defense^87^. Figure 12. depicts the process involved in GKSO.

### Mathematical formulation-hunting stage

Genghis khan type sharks conduct a broad space search near the unknown marine seashore. In the starting phase, the predator sharks travel in various directions till the position of the optimal food (prey) is located.

A random position near the prey is chosen by the shark which can be determined by computing the upper limits and lower limits. The superseding positions of the sharks are updated as per the following Eq. (2) as follows:

2 $${{Sh}_{i}}^{j+1}\left(t+1\right)={{Sh}_{i}}^{j}+\frac{{Lowerb}_{j}+rand*({Upperb}_{j}-{Lowerb}_{j})}{iter};\text{ i}=\text{1,2},3\dots \text{N};\text{ j}=\text{1,2},3\dots .\text{D};\text{ iter}=\text{1,2},3,\dots \text{T}$$

#### Movement stage

Once the prey is found the shark based on its olfactory senses tries to get closer enough to catch the prey. This movement of sharks can be defined as per the following Eq. (3) as follows:

3 $${{\widehat{Sh}}_{i}}^{j}\left(t+1\right)=\rho *\left({{sh}_{best}}^{j}\left(t\right)-{{sh}_{i}}^{j}\left(t\right)\right)$$

4 $$\rho =\mu {I}^{r}$$

From the Eq. (4), the value $\mu$ is mainly responsible for the convergence rate of the algorithm which is a positive constant. To find the optimal solution the value of $\mu$ has to be set as 1.5. The parameter ${I}^{r}$ denotes the Intensity attribute which is directly proportional to the capability of the self-sharks and $r$ is the abstract integer with the value ranging from 0 to 1. This value is directly proportional to the convergence rate of the sharks towards the odor.

When the value of $r=0$; no prey has been detected and the exploration phase of the shark continues. When $r=1$; it signifies the movement stage by absorbing the odor information of the self-sharks. If found the prey then it will be taken as the optimal solution. Sometimes when the value of $r=1$; relatively the value of $\rho$ also soars high. This implies that the algorithm supersedes the existing optimal solution and takes the new solution as the optimal solution thereby seizing exploration. The algorithm retains back the previous value of $r$ which denotes the optimal position of the prey and further updates of the remaining solutions is computed. The superseding of the position of the sharks is computed as per the Eq. (5) defined as follows:

5 $${{Sh}_{i}}^{j}\left(t+1\right)=\frac{{{\widehat{Sh}}_{i}}^{j}\left(t+1\right)+{{Sh}_{i-1}}^{j}\left(t\right)}{2}$$

#### Foraging stage

While hunting, the Genghis Khan-type sharks make a sudden shift in their travel exhibiting a parabolic-shaped attack which implies a cooperative hunting strategy. The foraging behaviour exhibited by the sharks is defined as per the Eq. (6) as follows:

6 $${{Sh}_{i}}^{j}\left(t+1\right)={{Sh}_{best}}^{j}\left(t\right)+rand* \left({{Sh}_{best}}^{j}\left(t\right)-{{Sh}_{i}}^{j}\left(t\right)\right)+\alpha * {\omega }^{2}*\left({{Sh}_{best}}^{j}\left(t\right)-{{Sh}_{i}}^{j}\left(t\right)\right)$$

The value $\alpha$ is taken as an abstract integer that lies between 1 to -1. The step size of the shark is impacted by the value of $\omega$ which is conducive to global exploration then falls to the local optima gradually. The value of $\omega \left(0\right)=1.0$ during the initialization stage. The formula to compute this factor $\omega$ is defined as follows:

7 $$\omega =2*\left\{1-{\left(\frac{t}{T}\right)}^\frac{1}{4}+\left|\cup \left(t+1\right)\right|*\left[{\left(\frac{t}{T}\right)}^\frac{1}{4}-{\left(\frac{t}{T}\right)}^{3}\right]\right\}$$

The value $\left|\cup \left(t+1\right)\right|$ at time t + 1 can be computed by using the Eq. (8) as follows:

8 $$\left|\cup \left(t+1\right)\right|=1-2{\omega }^{4}(t)$$

#### Self-defense stage

In case of any attack by the other marine creatures’ sharks lighten their body and perform an escaping pattern. This self-defense behaviour can be calculated using Eq. (9) as follows:

9 $$\left\{\begin{array}{c}{{Sh}_{i}}^{j}\left(t+1\right)={{Sh}_{i}}^{j}\left(t\right)+{k}_{1}\left({b}_{1}{{Sh}_{best}}^{j}\left(t\right)-{b}_{2}{{Sh}_{k}}^{j}\left(t\right)\right)+ {k}_{2}\cap \left({\complement }_{3}{{Sh2}_{i}}^{j}\left(t\right)-{{Sh1}_{i}}^{j}\left(t\right)\right)+\frac{{\cap }_{2}}{2}({{Sh}_{u1}}^{j}\left(t\right)-{{Sh}_{u2}}^{j}\left(t\right)); when {b}_{1}\le 0.5\\ {{Sh}_{i}}^{j}\left(t+1\right)={{Sh}_{i}}^{j}\left(t\right)+{k}_{1}\left({b}_{1}{{Sh}_{best}}^{j}\left(t\right)-{b}_{2}{{Sh}_{k}}^{j}\left(t\right)\right)+ {k}_{2}\cap \left({b}_{3}{{Sh2}_{i}}^{j}\left(t\right)-{{Sh1}_{i}}^{j}\left(t\right)\right)+\frac{{\cap }_{2}}{2}({{Sh}_{u1}}^{j}\left(t\right)-{{Sh}_{u2}}^{j}\left(t\right))\end{array}\right.$$

where $-1<{k}_{1}<1$ and ${k}_{2}$ pursues random distribution with a mean value of 0 and a standard deviation of 1. The computation of the random abstract attributes ${b}_{1}$, ${b}_{2}$, ${b}_{3}$ are defined in the manner described below:

10 $$\left\{\begin{array}{c}{b}_{1}={L}_{1}*2*rand+\left(1-{L}_{1}\right)\\ {b}_{2}={L}_{1}*rand+\left(1-{L}_{1}\right)\\ {b}_{3}={L}_{1}*rand+\left(1-{L}_{1}\right)\end{array}\right.$$

where ${L}_{1}\in \left[\text{0,1}\right]$ and $\cap$ is an adaptive coefficient that defines the relation between $\gamma$ and $\delta$.

11 $$\gamma =\left|\delta *\text{sin}\left(\frac{3\pi }{2}+\text{sin}\frac{3\pi \delta }{2}\right)\right|$$

12 $$\delta ={\delta }_{min}+\left({\delta }_{max}-{\delta }_{min}\right)*{\left({\left(1-\frac{iter}{Thresh}\right)}^{3}\right)}^{2}$$

13 $$\cap = \gamma *2*(rand-1)$$

The value of ${\delta }_{min}$=0.2 and ${\delta }_{max}=1$. The formula to compute ${{Sh1}_{i}}^{j}\left(t\right)$ and ${{Sh2}_{i}}^{j}\left(t\right)$ are defined as follows:

14 $${{Sh1}_{i}}^{j}\left(t\right)={Lowerb}_{j}+rand*\left({upperb}_{j}-{Lowerb}_{j}\right)$$

15 $${{Sh2}_{i}}^{j}\left(t\right)={Lowerb}_{j}+rand*\left({upperb}_{j}-{Lowerb}_{j}\right)$$

The formula to find the value of

16 $${{Sh}_{k}}^{j}\left(t\right)={L}_{2}*\left({{Sh}_{\omega }}^{j}\left(t\right)-{{Sh}_{\tau }}^{j}\left(t\right)\right)+{{Sh}_{\tau }}^{j}\left(t\right)$$

${{Sh}_{\omega }}^{j}\left(t\right)$ denotes the solutions of two random at time t ($\omega =\text{1,2},\dots N)$. Similarly, ${l}_{1},{l}_{2}\in \left[\text{0,1}\right]$ and ${{Sh}_{\tau }}^{j}\left(t\right)$ Implies the set of solutions generated randomly at time t. ${{Sh1}_{i}}^{j}\left(t\right)$ and ${{Sh2}_{i}}^{j}\left(t\right)$ are the selected random solutions. This provides the optimal solution by decomposing towards the optimal search space. This is similar to that of a neighbourhood search. This is a distinguishing feature or a property of GKSO which performs global exploration very efficiently.

#### Simulated annealing

Simulated annealing evolved by mimicking the behaviour of the metal being heated. It works based on the metal iron heating and cooling strategy. Let the temperature at the time ${t}_{0}$ represent the initial heat, and the temperature at ${t}_{f}$ represent the final temperature. The cooling rate is denoted as $\varphi$. A random set of solutions is generated. Which then gradually superseded by the upcoming solution prior to the previous solution. When the target temperature is met, then the algorithm gets terminated.

17 $${\text{Temp}} = {\text{Temp}}*\varphi ; \, \varphi \in \left[ {0,1} \right]$$

To alleviate the problem of local convergence, probabilities based on the worst solution can be accepted. The current temperature can be computed as.

$$e\left(\frac{-\Delta }{Temp}\right)\le rand$$ (18).

**Fig. 13:**
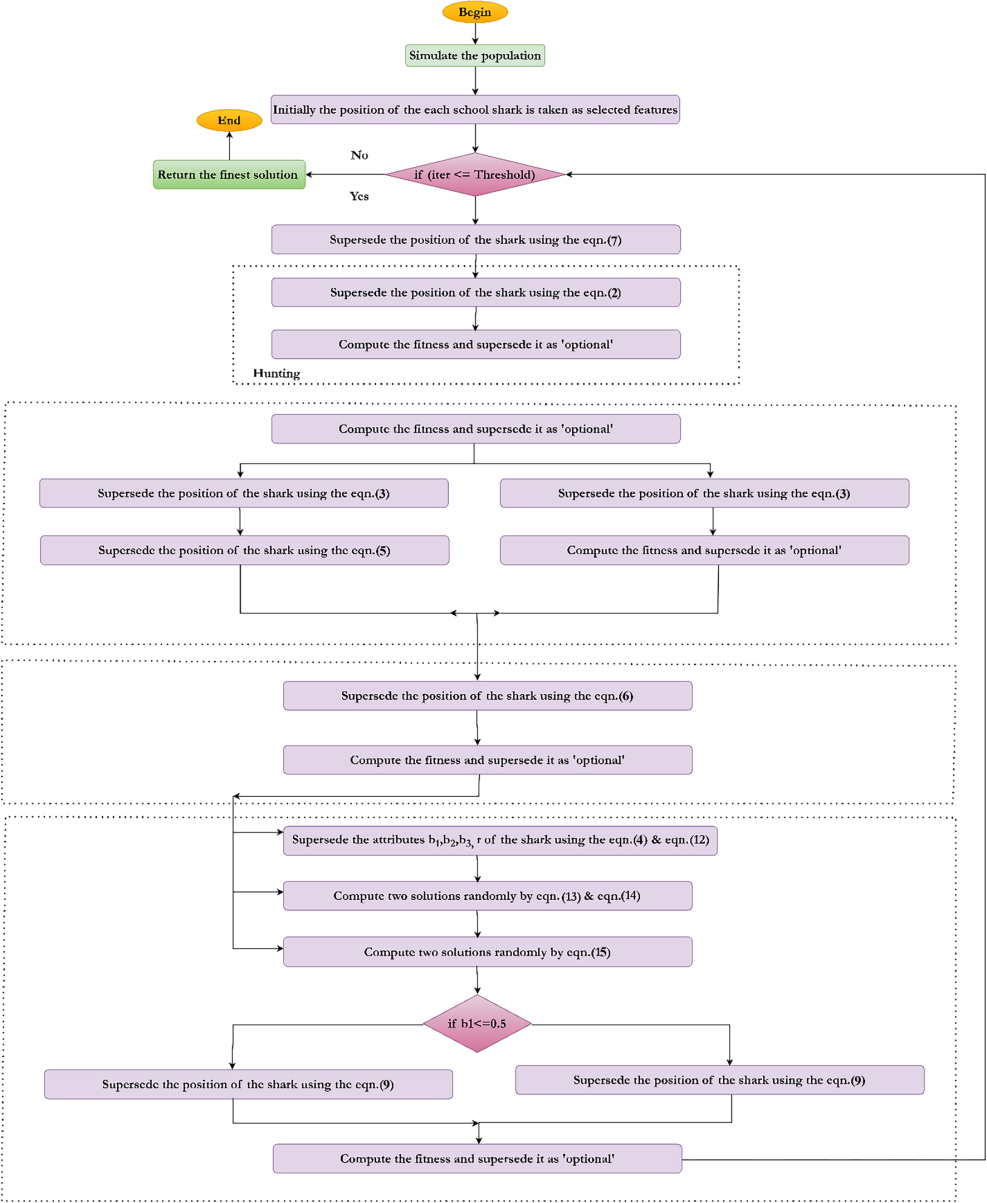
Hybridized GKSO-SA.

The factor $\Delta$ is the fitness function which is a difference between the current optimal to that of the previous optimal solution. The proposed hybridized GKSO-SA involves the steps depicted using Fig. 13. Algorithm 1 provides the working procedure of the hybridized GKSO-SA.

**Algorithm 1:**
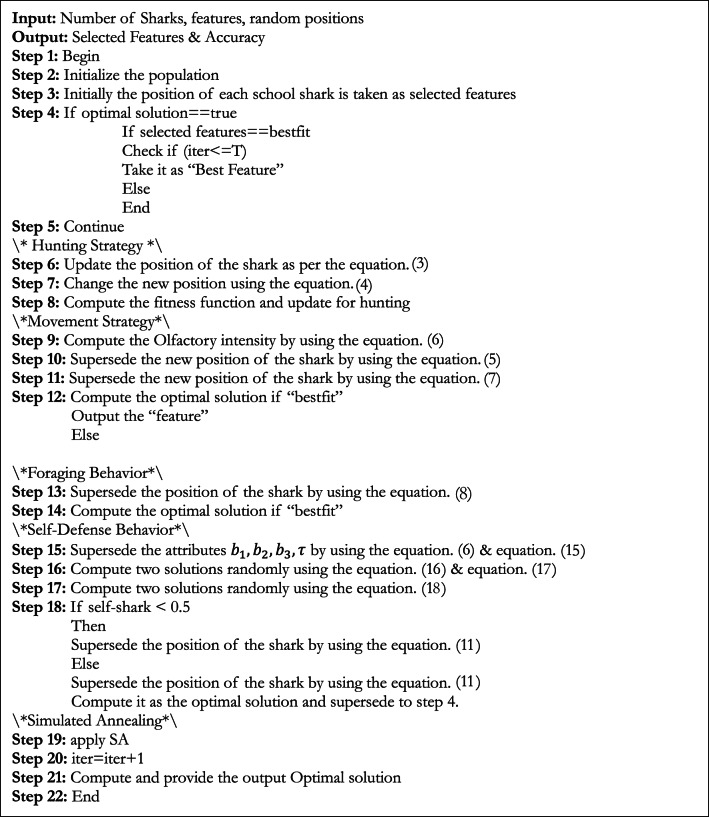
Hybrid GKSO-SA.

### Feature classification using CatBoost classifier

CatBoost classifiers possess advantages such as high classification performance, the capability to handle categorical data, and a robust regularization technique^88^. CatBoost is a potent ensemble learning-based predictive model that utilizes multiple decision trees (DT). The classifier generates a subsequent number of trees, which performs error corrections made by the previous tree^89^. Since the model maintains a symmetric tree organization, the output will not be affected by the order in which the features are given as input. The significant advantage of the CatBoost classifier is its performance by seizing the association between the categorical variable and the target variable^90^. The output obtained after classifying paddy diseased features will be either numerical (0 or 1) or binary (yes or no). A smoothing logloss function will be utilized for testing and validation^91^.

## Experimentation

This section presents the experimental setup, hardware and software configurations, dataset preparation, and evaluation metrics used to valid the proposed model.

### Implementation set-up

The proposed methodology was developed using Python^93^ and implemented on Google Colab^92^, leveraging libraries like Pandas, Keras, NumPy, and TensorFlow. The experiments were conducted using NVIDIA DGX GPU servers equipped with 512 GB of RAM and 8 high-speed Tesla V100 graphics proceeding units (GPUs), each with a capacity of 32 GB.

### Datasets used

Table 3. Distribution of diseases pertaining to paddy. The dataset utilized in the proposed methodology involves nine distinct kinds of paddy plant diseases such as blast, downy mildew, bacterial panicle blight, bacterial leaf blight, tungro, dead heart, bacterial leaf streak, hispa, and brown-spot obtained from the doctor paddy dataset. Paddy doctor dataset has been obtained from Kaggle^94^. The resulting dataset gets segregated into a set of training, testing, and validation sets in the ratio of 70:15:15.

**Table 3:** Presents the distribution of illnesses associated with paddy crops.

Disease	Class	Class no. (classification)		Image data			Total class-wise distribution (%)
		Binary	Multi-Class	Train	Test	Validate	
Bacterial leaf blight	Bacteria	0	0	478	100	100	678(6%)
Bacterial leaf streak		1	1	380	103	103	586(5%)
Bacterial panicle blight		2	2	337	90	90	516(5%)
Healthy Paddy	Fungus	3	3	588	123	123	834(8%)
Blast		0	4	1738	214	214	2166(11%)
Brown-spot		1	5	965	153	153	1271(11%)
Dead heart		2	6	1442	175	175	1792(12%)
Healthy Paddy		3	7	588	123	123	834(8%)
Hispa	Viral	0	8	1594	205	205	2004(10%)
Tungro		1	9	1088	188	188	1464(7%)
Downy mildew		2	10	620	133	133	886(8%)
Healthy Paddy		3	11	588 10,406	123 1730	123 1730	834(8%) 13,866(100%)

## Evaluation metrics

The effectiveness of the proposed hybrid model for classifying paddy leaf disease is evaluated using the following performance indicators:

i. *True Positives (TruePs)* The feature is classified as true positives when the classifier successfully labels it.
ii. *True Negatives (TrueNs)* True negatives are those features that fall under negative classification if the classifier deems them negative.
iii. *False Positives (FalsePs)* When the classifier incorrectly classifies then the feature belongs to True Negatives.
iv. *False Negatives (FalseNs)* False negatives are produced when the classifier classifies positive features as negative.
v. *Precision* This metric indicates the number of correctly identified positive samples, commonly known as the precision score.
  19 $$Precision \left(P\right)=\frac{TruePs}{TruePs+FalsePs}$$

**vi) Recall** This metric denotes the ratio of correctly identified positive samples to the total number of actual positive samples.

20 $$Recall \left(R\right)=\frac{TruePs}{FalseNs+TruePs}$$

**vii) Accuracy** This represents the proportion of correctly classified samples out of the total classifications.

21 $$Accuracy (Accy)=\frac{TruePs+TrueNs}{TruePs+TrueNs+FalsePs+FalseNs}$$

**viii) F1-measure** This value represents the balance between precision and recall, calculated as their harmonic mean

22 $$F1-Score=2*\frac{Precision*Recall}{Precision+Recall}$$

## Result analysis and discussion

The methodology has been implemented and analyzed for its performance in different machine learning and deep learning models, as well as existing systems, using metrics such as accuracy, precision, recall, and F1-score.

### Comparative analysis with various machine learning classification models

The proposed methodology has been classified against distinct ML classifiers like SVM, Random Forest, XGBoost, KNN, Naive Bayes, and CatBoost. The anticipated feature set was intended to enhance classifier performance, though it did increase the overall processing time required for the machine learning pipeline to complete. Table 4 displays the performance results of classifying the proposed methodology using different machine learning classifiers.

**Table 4:** Presents a comparison of various machine learning models.

ML Classifiers	Accuracy	Precision	Recall	F1-score
SVM	86.72	83.15	87.07	86.04
Random Forest	80.71	81.01	76.47	79.81
XGBoost	83.31	82.93	82.93	81.93
KNN	81.34	84.21	81.21	80.43
Naïve Bayes	79.24	77.47	77.47	76.12
CatBoost	92.6	89.12	93.21	92.13

**Fig. 14:**
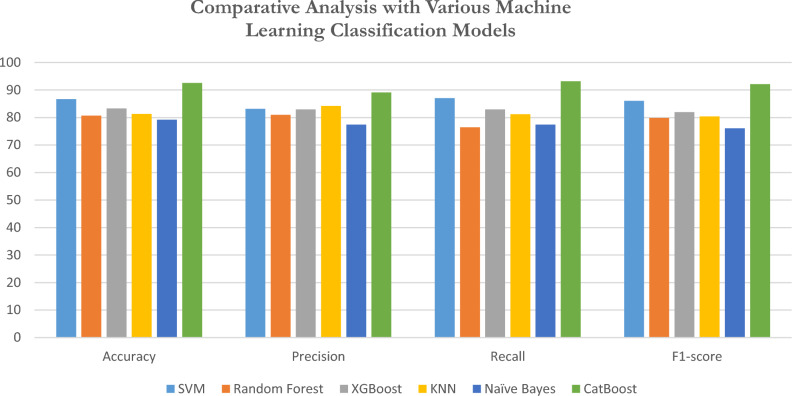
Shows a comparative analysis of various machine learning methods.

The proposed model, along with the deep features obtained from the MobileNetV3 + GKSO-SA + CatBoost classifier and other ML classifiers, as depicted in Fig. 14, demonstrates significant performance improvements. Support Vector Machine (SVM) reached an accuracy of 86.72%, with a precision of 83.15%, a recall of 87.07%, and an F1-score of 86.04%, indicating balanced performance across these metrics. Random Forest performed slightly lower, attaining an accuracy of 80.71%, precision of 81.01%, recall of 76.47%, and an F1-score of 79.81%, suggesting its suitability when precision is more important than recall. XGBoost delivered an accuracy of 83.31% and showed similar precision and recall values (82.93%), resulting in an F1-score of 81.93%, highlighting its consistency across precision and recall. KNN yielded an accuracy of 81.34%, the highest precision among these classifiers (84.21%), with recall at 81.21% and an F1-score of 80.43%, making it a reasonable choice when higher precision is required. Naïve Bayes demonstrated the lowest performance, with an accuracy of 79.24%, precision and recall at 77.47% and an F1-score of 76.12%, indicating limitations in handling complex datasets. CatBoost outperformed all other models, achieving an impressive accuracy of 92.6%, precision of 89.12%, recall of 93.21%, and an F1-score of 92.13%, making it an excellent choice for situations where both high accuracy and recall are essential.

### Comparative analysis with various deep learning classification models

The proposed methodology has been classified against different DL techniques such as Bi-GRU, LSTM, Bi-LSTM, CNN-Bi-GRU, CNN-LSTM, CNN-Bi-LSTM, CNN-GRU, and CNN. Table 5 displays the performance results of standard deep learning classifiers, comparing various deep learning models.

**Table 5:** Comparison of different deep learning models.

DL Classifiers	Accuracy	Precision	Recall	F1-score
CNN	96.22	97.99	96.1	94.22
LSTM	92.99	91.29	90.75	89.26
Bi-LSTM	97.55	96.42	95.28	94.66
Bi-GRU	90.81	92.54	91.88	92.11
CNN-LSTM	91.25	91.85	90.15	91.92
CNN-Bi-LSTM	95.18	94.48	93.21	92.12
CNN-GRU	93.95	93.45	92.45	91.21
CNN-Bi-GRU	94.25	94.81	93.73	90.75
MobileNetV3 + GKSO-SA	98.52	95.45	96.96	97.54

**Fig. 15:**
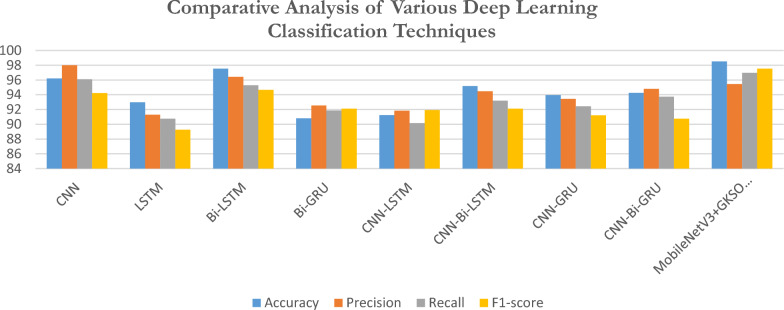
Comparative analysis of various deep learning methods.

Figure 15 presents a comparative analysis of various deep learning methods, where CNN achieved a high accuracy of 96.22%, with precision at 97.99%, recall at 96.1%, and an F1-score of 94.22%. This model displays a balanced and robust performance across all metrics, showing its ability to capture complex data patterns effectively. LSTM recorded an accuracy of 92.99%, with its precision, recall, and F1-score values slightly lower than CNN, at 91.29%, 90.75%, and 89.26%, respectively. While it performs well, LSTM may be more suited for tasks where moderate accuracy is acceptable, as it doesn’t quite reach the top performance observed in CNN and Bi-LSTM. Bi-LSTM stands out as the highest-performing model, recording an accuracy of 97.55%, precision of 96.42%, recall of 95.28%, and F1-score of 94.66%. The bidirectional characteristic improves its efficacy, rendering it appropriate for situations necessitating optimal accuracy and recall. Bi-GRU achieved an accuracy of 90.81% with a precision of 92.54%, a recall of 91.88%, and an F1-score of 92.11%. Although it performs well in precision and recall, its accuracy is slightly lower than CNN and Bi-LSTM. CNN-LSTM attained an accuracy of 91.25%, precision of 91.85%, recall at 90.15%, and an F1-score of 91.92%. This model offers a balanced performance, though it does not reach the highest accuracy scores seen in Bi-LSTM and CNN, making it a moderate choice. CNN-Bi-LSTM demonstrated an accuracy of 95.18%, with precision, recall, and F1-score values of 94.48%, 93.21%, and 92.12%, respectively. This combination shows strong performance and is especially beneficial for tasks that benefit from both CNN’s feature extraction and Bi-LSTM’s bidirectional processing. CNN-GRU achieved an accuracy of 93.95%, precision at 93.45%, recall at 92.45%, and an F1-score of 91.21%. While this model performs well, it doesn’t match the highest accuracy of Bi-LSTM or CNN, but it is still a practical choice in terms of balanced performance. CNN-Bi-GRU had an accuracy of 94.25%, with precision, recall, and F1-score values of 94.81%, 93.73%, and 90.75%, respectively. This model demonstrates vital precision and recall, although its F1-score is somewhat lower compared to other top-performing models.

### Comparative analysis of GKSO-SA versus other optimizers (PSO, GA, ACO)

This section provides a performance comparison between the proposed GKSO-SA hybrid optimization method and traditional metaheuristic algorithms, including Particle Swarm Optimization (PSO), Genetic Algorithm (GA), and Ant Colony Optimization (ACO), with emphasis on their effectiveness in feature selection and classification accuracy. Table 6 presents the performance results from a comparative analysis of the proposed GKSO-SA method against other optimization algorithms.

**Table 6:** Comparison of other optimization models.

Feature selector	Accuracy	Selected features	Time	Redundancy (%)	F1-score
PSO	95.13	89	125.4	6.9	94.62
GA	94.27	85	137.8	7.3	93.81
ACO	93.41	83	132.2	7.5	93.02
GKSO-SA	98.52	74	86.2	3.8	97.54

### Comparative analysis with various existing methodologies

The proposed methodology has classified the existing methods, such as Mbi-LSTM^23^, CNN-VGG^29^, DCNN^31^, IoT-CNN^33^, and GCN^34^. Table 7 shows the performance results from a comparative analysis of various existing methodologies.

**Table 7:** Comparative examination of different established methodologies.

Ref	Methods	Accuracy	Precision	Recall	F1-score
^23^	Mbi-LSTM	97.16	96.85	97.25	95.99
^29^	CNN-VGG	93	92.85	91.55	90.54
^31^	DCNN	96.08	95.52	94.45	95.75
^33^	IoT-CNN	97.7	96.13	95.55	96.55
^34^	GCN	97	96.55	95.25	94.32
Proposed Model	MobileNetV3 + GKSO-SA + CatBoost	98.52	95.45	96.96	97.54

**Fig. 16:**
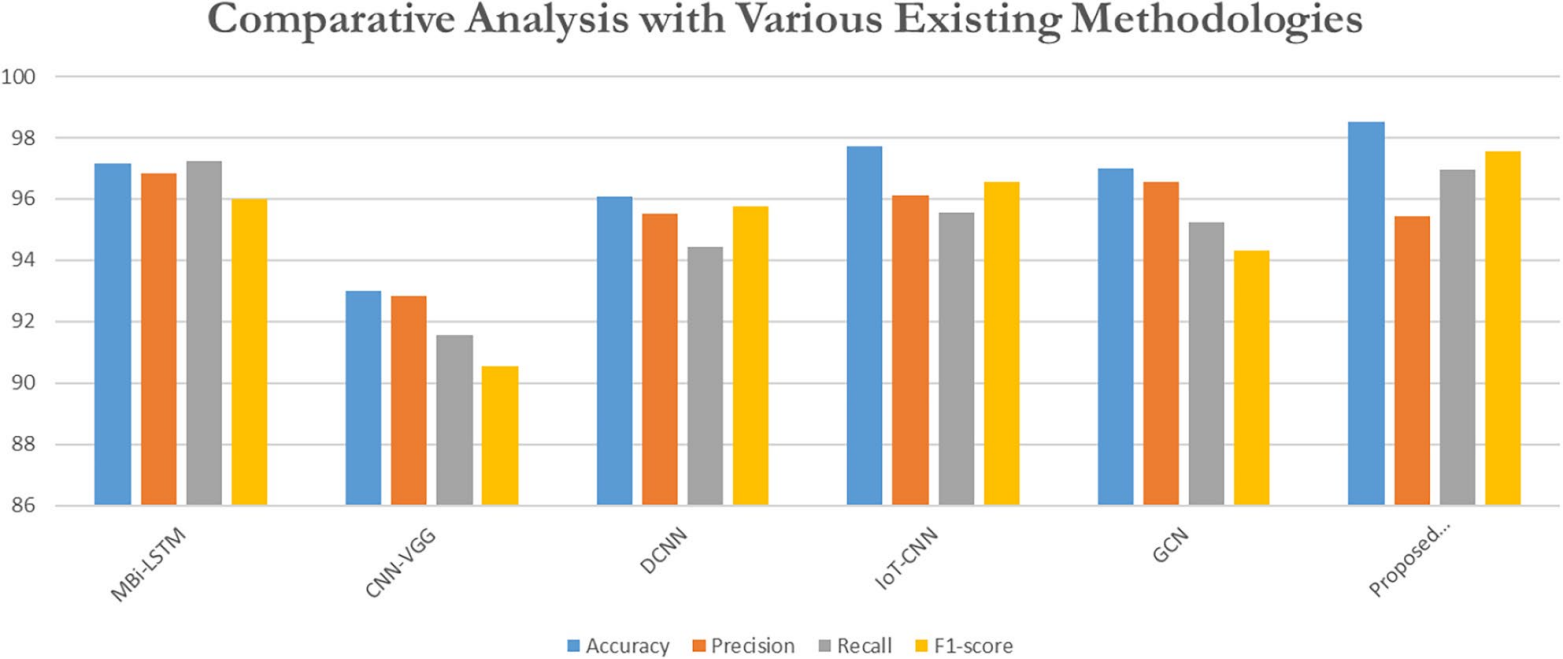
Performance evaluation of the proposed model in comparison to the previous approach.

Figure 16 presents the performance evaluation of the proposed model in comparison to the previous approach. A comparative analysis was conducted between the proposed model, which integrates MobileNetV3 with GKSO-SA (Genghis Khan Shark Optimizer with Simulated Annealing) and CatBoost, and several existing methodologies. The evaluation was based on metrics such as accuracy, precision, recall, and F1-score. Mbi-LSTM achieved an accuracy of 97.16%, with a precision of 96.85%, a recall of 97.25%, and an F1-score of 95.99%. This model demonstrates strong overall performance but needs to improve accuracy and F1-score compared to the proposed model. CNN-VGG reported an accuracy of 93%, precision of 92.85%, recall of 91.55%, and an F1-score of 90.54%. While this model performs well, its lower accuracy and F1 score highlight its limitations compared to more advanced or hybrid approaches. DCNN (Deep Convolutional Neural Network) showed an accuracy of 96.08%, with a precision of 95.52%, a recall of 94.45%, and an F1-score of 95.75%. DCNN performs effectively, though its accuracy and F1-score remain slightly lower than the proposed model’s. IoT-CNN achieved an accuracy of 97.7%, a precision of 96.13%, a recall of 95.55%, and an F1-score of 96.55%. This model demonstrates strong performance, particularly in the F1-score, but the proposed model surpasses it in accuracy. GCL recorded an accuracy of 97%, precision of 96.55%, recall of 95.25%, and an F1-score of 94.32%. GCN’s performance is solid, especially in precision, but it is still slightly outperformed by the proposed method in accuracy and F1-score. The proposed model (MobileNetV3 + GKSO-SA + CatBoost) stands out with the highest accuracy of 98.52%, precision of 95.45%, recall of 96.96%, and an F1-score of 97.54%. The combination of MobileNetV3’s lightweight architecture with the optimization capabilities of GKSO-SA and the robust classification power of CatBoost contributes to this model’s superior performance.

## Conclusion and future work

The research emphasizes the urgent need for efficient disease management strategies in rice farming, given the impact of plant diseases on both crop yield and quality. With increasing demand due to population growth, early and accurate detection of rice diseases is essential for timely intervention. The proposed automated deep learning model successfully enhances disease identification and categorization on paddy leaves by utilizing advanced image processing, feature extraction, and selection techniques. By utilizing MobileNetv3 for feature extraction and hybridizing Genghis Khan Shark Optimization (GKSO) with Simulated Annealing (SA) for optimal feature selection, the model attains a robust accuracy of 98.52% in classifying diseases. This highlights the model’s efficacy in improving agricultural productivity through deep learning technologies. Future work will focus on advancing this model by integrating Explainable AI (XAI) methods to enhance the transparency and interpretability of disease detection, enabling farmers to better comprehend the model’s predictions better.

Further enhancements may involve Artificial Specific Intelligence (ASI) techniques to create highly specialized models tailored for rice disease detection, ensuring even higher classification accuracy and efficiency. Exploring Artificial General Intelligence (AGI) could also expand the model’s adaptability, allowing it to autonomously generalize across various crop types and disease classifications. Together, these advancements could provide a more robust and adaptive tool for crop disease management.

## Data Availability

The datasets used and/or analyzed during the current study are available publicly and can be access with the link provide below. https://www.kaggle.com/competitions/paddy-disease-classification.

## References

[1] Han, H. & Lin, H. Patterns of agricultural diversification in China and its policy implications for agricultural modernization. *Int. J. Environ. Res. Public Health* **18**(9), 4978 (2021). 34067092 10.3390/ijerph18094978 PMC8125130

[2] Pugoy, R. A. D. & Mariano, V. Y. Automated rice leaf disease detection using color image analysis. In *Third International Conference on Digital Image Processing* vol. 8009 93–99 (SPIE, 2011).

[3] Naik, B. S., Shashikala, J. & Krishnamurthy, Y. L. ‘Study on the diversity of endophytic communities from rice (Oryza sativa L.) and their antagonistic activities in vitro’. *Microbiol. Res.* **164**(3), 290–296 (2009). 17433644 10.1016/j.micres.2006.12.003

[4] Jagan, M., Balasubramanian, M. & Palanivel, S. Detection and recognition of diseases from paddy plant leaf images. *Int. J. Comput. Appl.* **144**(12), 34–41 (2016).

[5] Phadikar, S., Sil, J. & Das, A. K. ‘Classification of rice leaf diseases based on morphological changes’. *Int. J. Inf. Electron. Eng.* **2**(3), 460–463 (2012).

[6] India: Yield of rice 1991–2021|Statista. https://www.statista.com/statistics/764299/india-yield-of-rice/. Accessed 16 Feb 2022

[7] Shrivastava, V. K., Pradhan, M. K., Minz, S. & Thakur, M. P. Rice plant disease classification using transfer learning of deep convolution neural network. *Int. Arch. Photogramm. Remote Sens. Spatial Inf. Sci.* **42**, 631–635 (2019).

[8] Leelavathy, B. & Rao Kovvur, R. M. Prediction of biotic stress in paddy crop using deep convolutional neural networks. In *Proceedings of International Conference on Computational Intelligence and Data Engineering* 337–346. 10.1007/978-981-15-8767-2_29 (Springer Singapore, 2020).

[9] Garg, G. et al. CROPCARE: an intelligent real time sustainable IoT system for crop disease detection using mobile vision. *IEEE Internet Things J.* **10**(4), 2840–2851 (2023).

[10] Bhola A, Kumar P (2023) Performance evaluation of different machine learning models in crop selection. In *Robotics, control and computer vision: select proceedings of ICRCCV 2022* 207–217 (Springer, 2023)

[11] Liu, K. & Zhang, X. PiTLiD: identification of plant disease from leaf images based on convolutional neural network. *IEEE/ACM Trans. Comput. Biol. Bioinform.* **20**(2), 1278–1288 (2022). 10.1109/TCBB.2022.3195291 35914052

[12] Subbarayudu, C. & Kubendiran, M. A comprehensive survey on machine learning and deep learning techniques for crop disease prediction in smart agriculture. *Nat. Environ. Pollut. Technol.* **23**(2), 619–632 (2024).

[13] Rao, U. S. et al. Deep learning precision farming: Grapes and mango leaf disease detection by transfer learning. *Global . Transit. Proc.* **2**(2), 535–544 (2021).

[14] Barburiceanu, S., Meza, S., Orza, B., Malutan, R. & Terebes, R. Convolutional neural networks for texture feature extraction. Applications to leaf disease classification in precision agriculture. *IEEE Access* **9**, 160085–160103 (2021).

[15] Bhavekar, G. S. & Goswami, A. D. A hybrid model for heart disease prediction using recurrent neural network and long short term memory. *Int. J. Inf. Technol.* **14**(4), 1781–1789 (2022).

[16] Haridasan, A., Thomas, J. & Raj, E. D. Deep learning system for paddy plant disease detection and classification. *Environ. Monit. Assess.* **195**(1), 120 (2023). 10.1007/s10661-022-10656-x 36399232

[17] Manikandan, G. et al. Classification models combined with Boruta feature selection for heart disease prediction. *Inform. Med. Unlock.* **44**, 101442 (2024).

[18] Puranik, S. S. et al. MobileNetV3 for mango leaf disease detection: an efficient deep learning approach for precision agriculture. In *2024 5th International Conference for Emerging Technology (INCET)* 1–7 (IEEE, 2024)..

[19] Hu, G., Guo, Y., Wei, G. & Abualigah, L. Genghis Khan shark optimizer: a novel nature-inspired algorithm for engineering optimization. *Adv. Eng. Inform.* **58**, 102210 (2023).

[20] Harish, M. et al. analysis on early prediction of cotton plant leaf diseases Using CatBoost algorithm. In *2024 2nd International Conference on Sustainable Computing and Smart Systems (ICSCSS)* 1458–1466 (IEEE, 2024).

[21] Bhola, A. & Kumar, P. Deep feature-support vector machine-based hybrid model for multi-crop leaf disease identification in Corn, Rice, and Wheat. *Multimed. Tools Appl.* **2024**, 1–21 (2024).

[22] Omer, S. M., Ghafoor, K. Z. & Askar, S. K. Lightweight improved yolov5 model for cucumber leaf disease and pest detection based on deep learning. *SIViP* **18**(2), 1329–1342 (2024).

[23] Dubey, R. K. & Choubey, D. K. Adaptive feature selection with deep learning MBi-LSTM model-based paddy plant leaf disease classification. *Multimed. Tools Appl.* **83**(9), 25543–25571 (2024).

[24] Elfatimi, E., Eryiğit, R. & Elfatimi, L. Deep multi-scale convolutional neural networks for automated classification of multi-class leaf diseases in tomatoes. *Neural Comput. Appl.* **36**(2), 803–822 (2024).

[25] Wang, B., Yang, H., Zhang, S. & Li, L. Identification of multiple diseases in apple leaf based on optimized lightweight convolutional neural network. *Plants* **13**(11), 1535 (2024). 38891344 10.3390/plants13111535 PMC11174786

[26] Stephen, A., Punitha, A. & Chandrasekar, A. Optimal deep generative adversarial network and convolutional neural network for rice leaf disease prediction. *Vis. Comput.* **40**(2), 919–936 (2024).

[27] Singh, A., Kaur, J., Singh, K. & Singh, M. L. Deep transfer learning-based automated detection of blast disease in paddy crop. *SIViP* **18**(1), 569–577 (2024).

[28] Dubey, R. K. & Choubey, D. K. An efficient adaptive feature selection with deep learning model-based paddy plant leaf disease classification. *Multimed. Tools Appl.* **83**(8), 22639–22661 (2024).

[29] Dogra, R. et al. Deep learning model for detection of brown spot rice leaf disease with smart agriculture. *Comput. Electr. Eng.* **109**, 108659 (2023).

[30] Bharanidharan, N. et al. Multiclass paddy disease detection using filter based feature transformation technique. *IEEE Access* (2023).

[31] Latif, G., Abdelhamid, S. E., Mallouhy, R. E., Alghazo, J. & Kazimi, Z. A. Deep learning utilization in agriculture: Detection of rice plant diseases using an improved CNN model. *Plants* **11**(17), 2230 (2022). 36079612 10.3390/plants11172230 PMC9460897

[32] Sethy, P. K., Barpanda, N. K., Rath, A. K. & Behera, S. K. Deep feature-based rice leaf disease identification using support vector machine. *Comput. Electron. Agric.* **175**, 105527 (2020).

[33] Debnath, O. & Saha, H. N. An IoT-based intelligent farming using CNN for early disease detection in rice paddy. *Microprocess. Microsyst.* **94**, 104631 (2022).

[34] Lamba, S., Baliyan, A. & Kukreja, V. A novel GCL hybrid classification model for paddy diseases. *Int. J. Inf. Technol.* **15**(2), 1127–1136 (2023). 36159716 10.1007/s41870-022-01094-6 PMC9484355

[35] Upadhyay, N. & Gupta, N. Detecting fungi-affected multi-crop disease on heterogeneous region dataset using modified ResNeXt approach. *Environ. Monit. Assess.* **196**(7), 610 (2024). 38862723 10.1007/s10661-024-12790-0

[36] Upadhyay, N. & Gupta, N. Mango crop maturity estimation using meta-learning approach. *J. Food Process Eng.* **47**(6), e14649 (2024).

[37] Upadhyay, N. & Gupta, N. Potato leaves disease detection with data augmentation using deep learning approach. In *International Conference on Information and Communication Technology for Competitive Strategie*s 589–599 (Springer Nature Singapore, 2022).

[38] Upadhyay, N. & Gupta, N. Diagnosis of fungi affected apple crop disease using improved ResNeXt deep learning model. *Multimed. Tools Appl.* **83**(24), 64879–64898 (2024).

[39] Upadhyay, N. & Gupta, N. A survey on diseases detection for agriculture crops using artificial intelligence. In *2021 5th International conference on Information Systems and Computer Networks (ISCON)* 1–8 (IEEE, 2021).

[40] Paddy Doctor: Paddy Disease Classification | Kaggle. Accessed: Jun. 25, 2024. [Online]. https://www.kaggle.com/competitions/paddy-disease-classification.

[41] Naqvi, S. A. H. Bacterial leaf blight of rice: An overview of epidemiology and management with special reference to Indian sub-continent. *Pak. J. Agric. Res.* **32**(2), 359 (2019).

[42] Wahab, W. A. et al. Disease development and discovery of anatomically resistant features towards bacterial leaf streak in rice. *Agriculture* **12**(5), 629 (2022).

[43] Mulaw, T., Wamishe, Y. & Jia, Y. Characterization and in plant detection of bacteria that cause bacterial panicle blight of rice. *Am. J. Plant Sci.* **9**(4), 667–684 (2018).

[44] Lamba, S., Kukreja, V., Baliyan, A., Rani, S. & Ahmed, S. H. A novel hybrid severity prediction model for blast paddy disease using machine learning. *Sustainability* **15**(2), 1502 (2023).

[45] Sunder, S., Singh, R. A. M. & Agarwal, R. Brown spot of rice: An overview. *Indian Phytopathol* **67**(3), 201–215 (2014).

[46] Tripathi, P. P., Anup, C. & Asha, S. Suppression of dead-heart and folded leaf symptoms in paddy by Trichogramma japonicum Ashmead in Seppa area of Arunachal Pradesh. *India. Environ. Ecol* **35**, 1297–1299 (2017).

[47] Thakur, R. P. & Mathur, K. Downy mildews of India. *Crop Prot.* **21**(4), 333–345 (2002).

[48] Ali, M. A., Sharma, A. K. & Dhanaraj, R. K. Heterogeneous features and deep learning networks fusion-based pest detection, prevention and controlling system using IoT and pest sound analytics in a vast agriculture system. *Comput. Electr. Eng.* **116**, 109146 (2024).

[49] Sharma, M. & Kumar, C. J. Improving rice disease diagnosis using ensemble transfer learning techniques. *Int. J. Artif. Intell. Tools* **31**(08), 2250040 (2022).

[50] Liu, J., Zhu, F., Chai, C., Luo, Y. & Tang, N. Automatic data acquisition for deep learning. *Proc. VLDB Endowment* **14**(12), 2739–2742 (2021).

[51] Nagaraju, M. et al. Systematic review of deep learning techniques in plant disease detection. *Int. J. Syst. Assur. Eng. Manag.* **11**, 547–560 (2020).

[52] Shorten, C. & Khoshgoftaar, T. M. A survey on image data augmentation for deep learning. *J. Big Data* **6**, 1–48 (2019). 10.1186/s40537-021-00492-0 PMC8287113 34306963

[53] Lopes, L. A., Machado, V. P., Rabelo, R. A., Fernandes, R. A. & Lima, B. V. Automatic labelling of clusters of discrete and continuous data with supervised machine learning. *Knowl.-Based Syst.* **106**, 231–241 (2016).

[54] Thomas, J. & Raj, E. D. Effectual single image dehazing with color correction transform and dark channel prior. In *International Conference on Information Processing* 29–41 (Springer, 2021).

[55] Khirade, S. D. & Patil, A. B. Plant disease detection using image processing. In *2015 International Conference on Computing Communication Control and Automation* 768–771 (2015).

[56] Chaki, J. & Dey, N. *A Beginner’s Guide to Image Shape Feature Extraction Techniques* (CRC Press, 2019).

[57] Rosipal, R., Girolami, M., Trejo, L. J. & Cichocki, A. Kernel PCA for feature extraction and de-noising in nonlinear regression. *Neural Comput. Appl.* **10**, 231–243 (2001).

[58] Sony, S., Dunphy, K., Sadhu, A. & Capretz, M. A systematic review of convolutional neural network-based structural condition assessment techniques. *Eng. Struct.* **226**, 111347 (2021).

[59] Lu, J., Tan, L. & Jiang, H. Review on convolutional neural network (CNN) applied to plant leaf disease classification. *Agriculture* **11**, 707 (2021).

[60] Joppa, L. N. *The Case for Technology Investments in the Environment* (2017). 10.1038/d41586-017-08675-7 32094673

[61] Nixon, M. & Aguado, A. *Feature Extraction and Image Processing for Computer Vision* (Academic Press, 2019).

[62] Barga, R., Fontama, V., & Tok, W. H. Introducing Microsoft Azure machine learning. In *Predictive Analytics with Microsoft Azure Machine Learning* 21–43 (Springer, 2015).

[63] Barga, R., Fontama, V., Tok, W. H. & Cabrera-Cordon, L. *Predictive Analytics with Microsoft Azure Machine Learning* (Springer, 2015).

[64] Brownlee, J. Deep learning for computer vision: image classification, object detection, and face recognition in python. *Machine Learning Mastery* (2019).

[65] Benesty, J., Chen, J. & Huang, Y*. Classical Optimal Filtering. Microphone Array Signal Processing* 7–37 (2008).

[66] Ilesanmi, A. E., Idowu, O. P., Chaumrattanakul, U. & Makhanov, S. S. Multiscale hybrid algorithm for pre-processing of ultrasound images. *Biomed. Signal Process. Control* **66**, 102396. 10.1016/j.bspc.2020.102396 (2021).

[67] Chandra, T. B. & Verma, K. Analysis of quantum noise reducing filters on chest X-ray images: A review. *Measurement* **153**, 107426. 10.1016/j.measurement.2019.107426 (2020).

[68] Thangadurai, K. & Padmavathi, K. Computer vision image enhancement for plant leaves disease detection. In *2014 World Congress on Computing and Communication Technologies* 173–175 (IEEE, 2014).

[69] Mikołajczyk, A. & Grochowski, M. Data augmentation for improving deep learning in image classification problem. In *2018 international Interdisciplinary PhD Workshop (IIPhDW)* 117–122 (IEEE, 2018).

[70] Kaur, D. & Kaur, Y. Various image segmentation techniques: a review. *Int. J. Comput. Sci. Mob. Comput.* **3**(5), 809–814 (2014).

[71] Saha, P. K. & Udupa, J. K. Optimum image thresholding via class uncertainty and region homogeneity. *IEEE Trans. Pattern Anal. Mach. Intell.* **23**(7), 689–706 (2001).

[72] Issac, A., Sarathi, M. P. & Dutta, M. K. An adaptive threshold based image processing technique for improved glaucoma detection and classification. *Comput. Methods Programs Biomed.* **122**(2), 229–244 (2015). 26321351 10.1016/j.cmpb.2015.08.002

[73] Lofroth, M. & Avci, E. Auto-focusing approach on multiple micro objects using the prewitt operator. *Int. J. Intell. Robot. Appl.* **2**(4), 413–424 (2018).

[74] Jumb, V., Sohani, M. & Shrivas, A. Color image segmentation using K-means clustering and Otsu’s adaptive thresholding. *Int. J. Innov. Technol. Explor. Eng.* **3**(9), 72–76 (2014).

[75] Mairal, J., Koniusz, P., Harchaoui, Z. & Schmid, C. Convolutional kernel networks. *Adv. Neural Inf. Process. Syst.* **27**, 332 (2014).

[76] Ahmed, A. S. Comparative study among Sobel, Prewitt and Canny edge detection operators used in image processing. *J. Theor. Appl. Inf. Technol* **96**(19), 6517–6525 (2018).

[77] Abildayeva, T. & Shamoi, P. Fuzzy logic approach for visual analysis of websites with K-means clustering-based color extraction. *arXiv preprint*. arXiv:2408.00774 (2024).

[78] Mustafa, W. A., Khairunizam, W., Ibrahim, Z., Shahriman, A. & Razlan, Z. M. A review of different segmentation approach on non uniform images. In *2018 International Conference on Computational Approach in Smart Systems Design and Applications (ICASSDA)* 1–6 (IEEE, 2018).

[79] Yin, X., Li, W., Li, Z. & Yi, L. Recognition of grape leaf diseases using MobileNetV3 and deep transfer learning. *Int. J. Agric. Biol. Eng.* **15**(3), 184–194 (2022).

[80] Abdel-Salam, M., Alzahrani, A. I., Alblehai, F., Zitar, R. A. & Abualigah, L. An improved Genghis Khan optimizer based on enhanced solution quality strategy for global optimization and feature selection problems. *Knowl.-Based Syst.* **302**, 112347 (2024).

[81] Ibrahim, H. T., Mazher, W. J. & Yaseen, Z. F. Hybrid feature selection approach based on firefly algorithm and simulated annealing for cancer datasets. *Univ. Thi-Qar J. Eng. Sci.* **14**(1), 1–9 (2024).

[82] Thota, K. K. et al. A Model for Predicting Chronic Renal Failure using CatBoost Classifier Algorithm and XGBClassifier. In *2024 Second International Conference on Inventive Computing and Informatics (ICICI)* 96–102 (IEEE, 2024).

[83] Modak, S. K. S. & Jha, V. K. Diabetes prediction model using machine learning techniques. *Multimed. Tools Appl.* **83**(13), 38523–38549 (2024).

[84] Elaziz, M. A., Dahou, A., El-Sappagh, S., Mabrouk, A. & Gaber, M. M. AHA-AO: Artificial hummingbird algorithm with aquila optimization for efficient feature selection in medical image classification. *Appl. Sci.* **12**, 9710. 10.3390/app12199710 (2022).

[85] Ramachandran, P., Zoph, B. & Le, Q.V. 2017 Searching for activation functions. *arXiv*. arXiv:1710.05941.

[86] Elfwing, S., Uchibe, E. & Doya, K. Sigmoid-weighted linear units for neural network function approximation in reinforcement learning. *Neural Netw.* **107**, 3–11 (2018). 29395652 10.1016/j.neunet.2017.12.012

[87] Wang, C., Yao, X., Ding, F. & Yu, Z. A trajectory planning method for a casting sorting robotic arm based on a nature-inspired Genghis Khan shark optimized algorithm. *Math. Biosci. Eng.* **21**(2), 3364–3390 (2024). 38454732 10.3934/mbe.2024149

[88] Prokhorenkova, L., Gusev, G., Vorobev, A., Dorogush, A. V. & Gulin, A. CatBoost: Unbiased boosting with categorical features. *Adv. Neural Inf. Process. Syst.* **31**, 1–11 (2018).

[89] Dorogush, A. V., Ershov, V. & Gulin, A. CatBoost: Gradient boosting with categorical features support. *ArXiv Preprint* arXiv:1810.11363 (2018).

[90] Hancock, J. T. & Khoshgoftaar, T. M. Survey on categorical data for neural networks. *J. Big Data* **7**(1), 28. 10.1186/s40537-020-00305-w (2020). 10.1186/s40537-020-00369-8 PMC7610170 33169094

[91] Hao, D., Xiaoqi, Y. & Taoyu, Q. Hybrid machine learning models based on CATBoost classifier for assessing students’ academic performance. *Int. J. Adv. Comput. Sci. Appl.* **15**(7), 94–106 (2024).

[92] Asmar, E., Vahidnia, M. H., Rezaei, M. & Amiri, E. Remote sensing-based paddy yield estimation using physical and FCNN deep learning models in Gilan province, Iran. *Remote Sens. Appl.* **34**, 101199 (2024).

[93] Jeyanathan, J. S. et al. Pesticide Recommender System for Detecting the Paddy Crop Diseases through SVM. In *2024 Third International Conference on Intelligent Techniques in Control, Optimization and Signal Processing (INCOS)* 1–6 (IEEE, 2024).

[94] Babu, B. A., & Dass, P. Detection of disease in fresh fruits using convolution neural network by comparing with KNN to maximize the accuracy and sensitivity. In *AIP Conference Proceedings* Vol. 2853, No. 1) (AIP Publishing, 2024).

